# Platinum prodrug nanoparticles inhibiting tumor recurrence and metastasis by concurrent chemoradiotherapy

**DOI:** 10.1186/s12951-022-01322-y

**Published:** 2022-03-12

**Authors:** Wei Jiang, Lulu Wei, Bing Chen, Xingyu Luo, Peipei Xu, Jianfeng Cai, Yong Hu

**Affiliations:** 1 Department of Hematology, Nanjing Drum Tower Hospital, Nanjing University, Nanjing, 210093 China; 2 Institute of Materials Engineering, College of Engineering and Applied Sciences, Nanjing University, Jiangsu, 210093 China; 3 Department of Chemistry, University of South Florida, 4202 E. Fowler Ave, Tampa, FL 33620 USA

**Keywords:** Concurrent chemoradiotherapy, Platinum prodrug, Ferroptosis, Tumor recurrence, Tumor metastasis

## Abstract

**Background:**

Although concurrent chemoradiotherapy (CRT), as one of the most effective antineoplastic therapies in clinic, can successfully inhibit the growth of tumor cells, a risk of developing secondary tumor is still an insurmountable barrier in clinical practice.

**Results:**

Herein, a new platinum prodrug composed of tannic acid (TA) and Pt^2+^ (TA-Pt) complex film was synthesized on the surface of Fe_2_O_3_ nanoparticles (NPs) with excellent stability and biocompatibility for enhanced CRT. In this system, TA-Pt complex could respond to the tumor acidic microenvironment and damage the DNA of tumor cells. Moreover, the internal iron core not only improved the effect of subsequent radiotherapy (RT), but also disrupted the iron balance in cells, inducing intracellular ferroptosis and eliminating apoptosis-resistant cells. In vitro and vivo experimental results indicated that more than 90% of tumor cells were depleted and more than 75% of the cured tumor-bearing mice evinced no recurrence or metastasis.

**Conclusions:**

This work offered a new idea for combining the effective chemotherapy, RT and ferroptosis therapy to enhance the curative effect of CRT and inhibit tumor recurrence and metastasis.

**Graphical Abstract:**

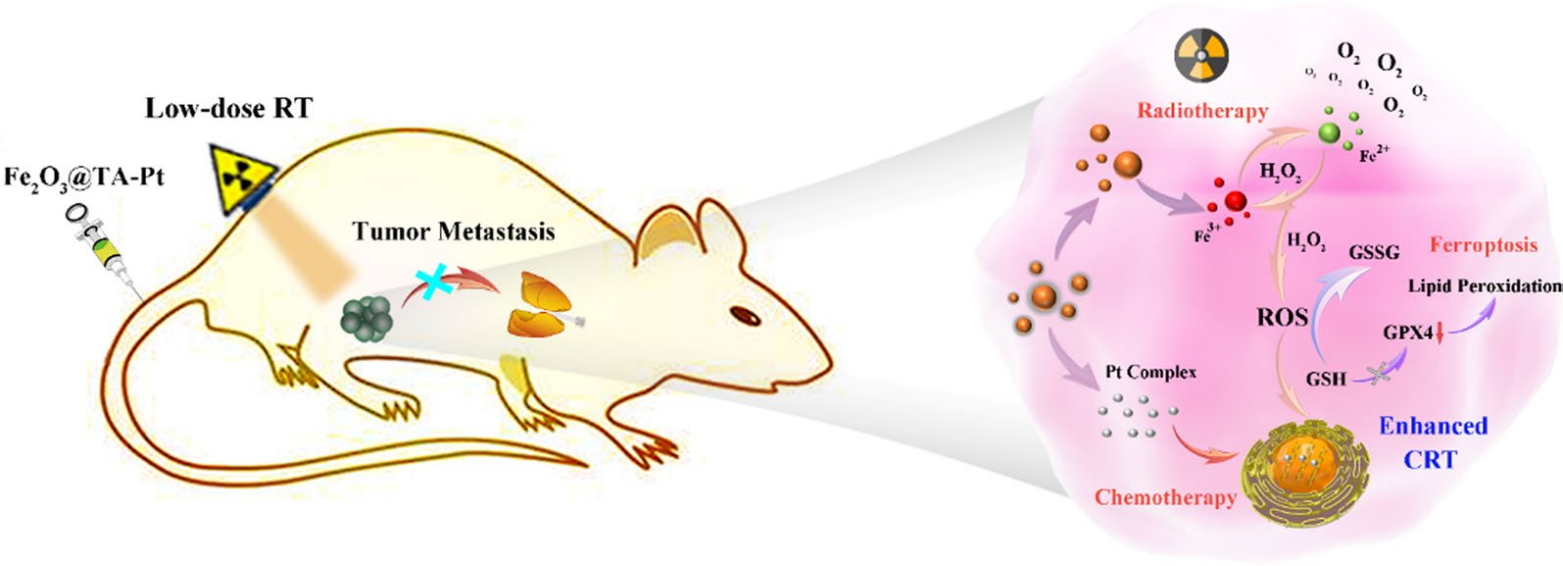

**Supplementary Information:**

The online version contains supplementary material available at 10.1186/s12951-022-01322-y.

## Introduction

In clinic, chemotherapy with excellent applicability and antitumor activity has been widely applied in various antineoplastic therapy [1–5]. Specifically, more than 50% of patients choose to use platinum-based chemotherapeutic drugs, which can be embed in DNA chains, and inhibit the tumor cell proliferation by preventing DNA from being copied and transcribed [6]. However, inevitable drug resistance and high nephrotoxicity have been confirmed in the process of adopting platinum-based chemotherapy drugs for oncotherapy [7–10]. Moreover, nausea, vomiting, and diarrhea are common complications of platinum-based drug therapy, the application of platinum-based chemotherapeutic drugs in clinical antitumor therapy has been greatly restricted [11–13]. Therefore, the low dose platinum-based drugs and radiotherapy (RT) are often combined for clinical concurrent chemoradiotherapy (CRT) against tumors.

CRT, a tumor treatment method combining chemotherapy and radiotherapy to increase radiotherapy effect and inhibit tumor cell growth, has been verified clinically to improve the survival of cancer patients. But even though great achievements of CRT have been made in the recent years, tumor recurrence and metastasis are still insurmountable barriers in clinical practice, nearly 60% of malignant tumor patients suffer from tumor recurrence or metastasis within one year post CRT [14–20]. Generally, RT mainly induces necrosis and apoptosis of tumor cells, accompanied by some side effects including the damage to surrounding normal tissues and the immune system, especially with high dose of radiation [21–23]. Recently, it has discovered that vast necrosis within tumor cells always leads to the generation of excessive inflammatory factors, thus accelerating the recurrence and metastasis of the tumor [24–26]. Moreover, around 30% of existing cancers express mutated small GTPases, which presents an endogenous inhibition on apoptosis pathways, thus leading to a severe drug resistance in apoptosis-induced tumor therapy in malignant tumors [27–29]. Therefore, improving the therapeutic effect of CRT and adopting an adequate treatment other than apoptosis with less tumor recurrence and metastasis are still highly desired.

**Scheme 1:**
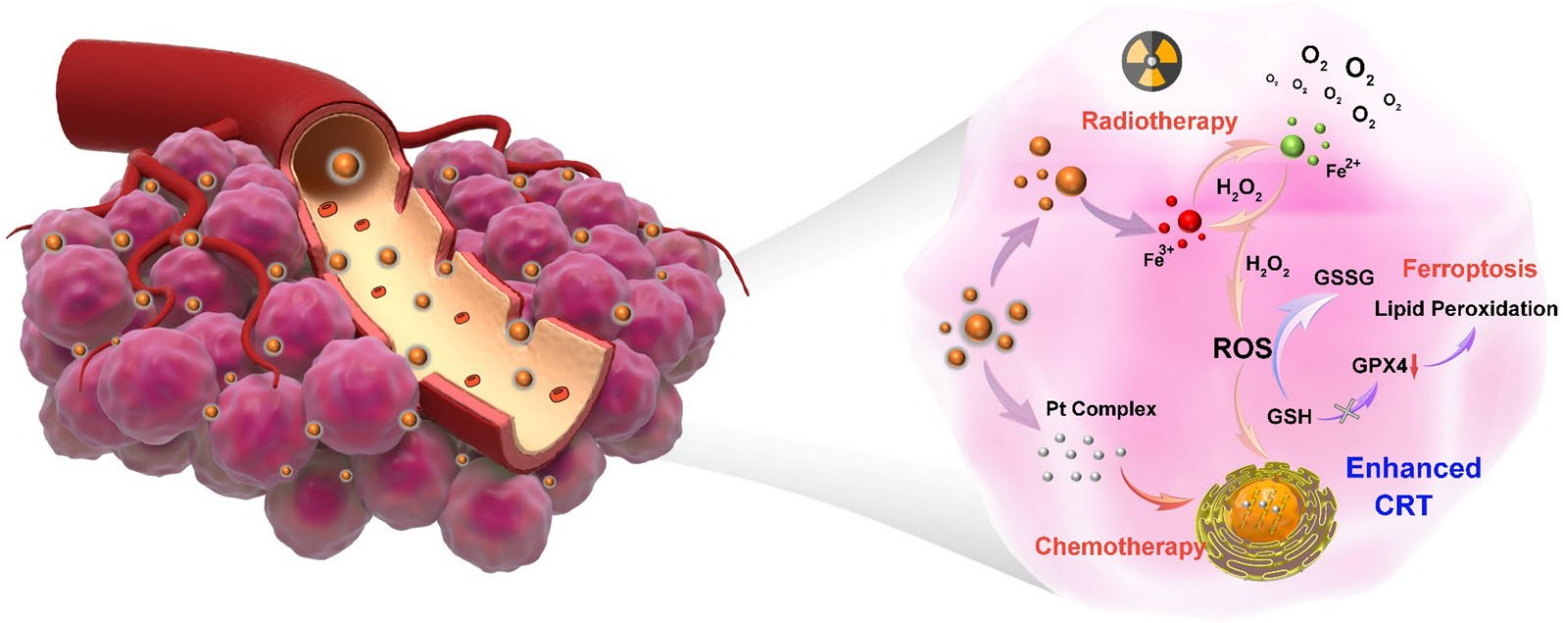
Schematic illustration of Fe_2_O_3_@TA-Pt NPs and their anti-tumor mechanism

Ferric oxide composite is considered to be one of the most promising nanomaterials for clinical oncotherapy due to its excellent biocompatibility [30–32]. In the process of tumor therapy, ferric oxide composite can increase the RT effect through the secondary radiation effect as well as breaking the iron balance in tumor cells to induce intracellular ferroptosis, which can destroy apoptosis-resistant tumor cells [33]. With this regard, we rationally synthesized a new platinum prodrug by coordinating tannic acid (TA) and Pt^2+^ to form TA-Pt complex film on the surface of Fe_2_O_3_ nanoparticles (NPs). In this system, the iron core with controllable morphology was synthesized by a simple method. TA-Pt film can not only improve the hydrophilicity and stability of Fe_2_O_3_ core, but also enrich the content of Fe_2_O_3_ in tumor site through the enhanced permeability and retention (EPR) effect. In response to the acidic tumor environment, TA-Pt film was partially disintegrated and embedded inside DNA acted as chemotherapeutic agents to prevent tumor cell proliferation. More importantly, Fe_2_O_3_ core provided a stable and sufficient iron supply to disrupt intracellular iron balance for ferroptosis therapy, and accompanied by the generation of an abundant of ROS and O_2_ for enhanced CRT (Scheme 1). In combination with chemotherapy, low dose RT and ferroptosis, tumor cells could be well eliminated, restricting the tumor recurrence and metastasis with less side effects. Collectively, this nanoplatform (Fe_2_O_3_@TA-Pt NPs) offers a potential CRT to conquer malignant tumors.

## Methods

### Materials

Iron nitrate (Fe(NO_3_)_3_, 98.5%), sodium chloride (NaCl, 99.5%), tannic acid (TA, 95%), potassium tetrachloroplatinate (II) (K_2_PtCl_4_, 99.99%) and 5,5-Dimethyl-1-pyrroline *N*-oxide (DMPO, 97%) were supplied from J&K Science (Beijing, China). Hydrogen peroxide (H_2_O_2_, 30%) was purchased from Shanghai Lingfeng Chemical Reagent Co., Ltd. (Shanghai, China). Polyethylene glycol (5000) cyanine 5.5 (OH-PEG-Cy5.5) and polyethylene glycol fluorescein isothiocyanate (OH-PEG-FITC) were obtained from Hunan Hua Teng Pharmaceutical Co., Ltd. All chemicals were used as received without further purification.

### Cell lines

Mouse Breast Cancer cells (4T1) and Human Umbilical Vein Edothelial cells (HUVEC) were supplied by the Shanghai Institute of Cell Biology (Shanghai, China). Roswell Park Memorial Institute 1640 (RPMI-1640) medium, trypsin-EDTA (0.25%), phosphate buffered saline (PBS, pH = 7.4, 6.8, 5.5) and 4′,6-diamidino-2-phenylindole (DAPI) were purchased from KeyGEN BioTECH (Nanjing, China). Endothelial Cell Growth Medium-2 (EGM-2) was supplied from Lonza (Switzerland). Cell Counting Kit-8 (CCK-8), LysoTracker Red DND-99, hydrogen peroxide reagent kit, GSH Assay kit and DNA extraction kit were supplied from Beyotime (Shanghai, China). Hypoxia/Oxidative stress detection kit was purchased from Enzo Life Sciences (New York, America). Fetal bovine serum (FBS) was purchased from Bio-Sciences Limited (Irish). BODIPY^581/591^-C11, *N*-acetylcysteine (NAC), GPX4 Recombinant Rabbit Monoclonal Antibody, beta Actin Monoclonal Antibody and Goat anti-Rabbit IgG (H + L) Secondary Antibody (HRP) were purchased from Thermo Fisher Scientific Incorporated (Waltham, America). Phalloidin-iFluor 488 and Anti-gamma H2A.X (Alexa Fluor 647) were obtained from Abcam (United Kingdom). 4T1 cells were cultured in RPMI-1640 medium supplemented with 10% FBS in an incubator at 37 °C with 5% CO_2_ and HUVEC cells were cultured in EGM-2 under the same culture condition.

### Animal models

Balb/c mice (female, 6–8 weeks) were purchased from the Comparative Medicine Centre of Yangzhou University and raised in specific pathogen-free (SPF) facility. To set up the 4T1 tumor model, mice were inoculated subcutaneously with 4T1 cell line (1 × 10^6^ cells per mouse). All animal experiments were reviewed and approved by the Committee on Animals at Nanjing University and the guidance and support were provided by the National Institute of Animal Care.

### Preparation of FeOCl nanoparticles (FeOCl NPs) and Fe_2_O_3_ NPs

FeOCl NPs were synthesized by a simple meyhod. Briefly, 440 mg Fe(NO_3_)_3_ was added into 50 mL of deionized water, and the mixture was heated to boiling and kept refluxing until the suspension became limpid. After five minutes, 5 mL saturated saltwater was slowly injected into the reaction system drop by drop and a large amount of reddish-brown solid was produced in the meantime. After that, the solution was cooled to room temperature, and centrifuged at 16,000 rpm for 15 min. The FeOCl NPs were washed with deionized water for several times to remove the excess NaCl.

Then, 50 mg FeOCl NPs were re-dispersed in 30 mL of deionized water by ultrasonic and then transferred into a poly(tetrafluoroethylene) (Teflon)-lined autoclave (50 mL). The autoclave was placed in a muffler oven and heated to 200 °C for 24 h with a rate of 5 °C per minute. The final samples were collected by centrifugation at 16,000 rpm, purified by deionized water and dried by lyophilization.

For Fe_2_O_3_ NPs with small size, the initial content of Fe(NO_3_)_3_ was 44 mg, and other things being equal.

### Preparation of Fe_2_O_3_@TA-Pt NPs

20 mg Fe_2_O_3_ NPs was dispersed in 50 mL of deionized water. Then 100 µL of TA solution (40 mg/mL in deionized water) and 100 µL of K_2_PtCl_4_ solution (15 mg/mL in deionized water) were added into the suspension simultaneously. After overnight reaction, the solid samples were harvested by centrifuging at 16,000 rpm and washed with deionized water and absolute ethanol repeatedly. For synthesizing dye-label Fe_2_O_3_@TA-Pt NPs, 1 mg PEG-modified dye (OH-PEG-FITC or OH-PEG-Cy5.5) was added into the reaction system while adding TA and K_2_PtCl_4_.

### Characterizations

The morphology and size of the NPs were recorded by transmission electron microscopy (TEM, Model Tecnai 12, Philips Co., Ltd., Holland) and scanning electron microscopy (SEM, ΣIGMA, Zeiss, Germany). The elemental analysis was detected by sectional energy-dispersive spectroscopy (EDS). The Zeta potential was detected by Brookhaven Zataplus. Particle size and size distribution of these NPs were analyzed by dynamic light scattering (DLS, BI-9000AT, Brookhaven). X-ray diffraction (XRD) (λ = 1.54056 Å, Bruker Co., Ltd., Germany) was utilized to detect the crystalline phases of these samples. The hybrid bonding state of the samples were determined by X-ray photoelectron spectroscopy (XPS, Thermo Fisher K-Alpha, America). The UV-vis absorbance spectra of the products were measured by UV-vis spectrophotometry (UV3100, Shimadzu, Japan). The content of Pt in cells, organs and tumors was detected by inductively coupled plasma-mass spectrometer (ICP-MS; NexION 300 D, PerkinElmer Corporation, America). Hydroxyl radical was investigated with DMPO by spin-trapping EPR technique (Bruker EMXplus-10/12 spectrometer, Germany).

### Cytotoxicity assay

The cytotoxicity of these samples were monitored by CCK-8 test. Briefly, HUVEC and 4T1 cells were pre-seeded in the 96-well plates (5 × 10^3^ cells per well) and incubated at 37 °C overnight. Then, different concentrations of Fe_2_O_3_@TA-Pt NPs dispersed in fresh medium without FBS were adopted to replace the culture medium in each well. After that, cells were incubated at 37 °C for anthor 24 or 48 h. Then 10 µL of CCK-8 was added into each well and further incubated for several hours until the color of medium became orange. The absorbance of each well at 450 nm was measured with an iMark Enzyme mark instrument (Bio-Rad Inc., America).

### Intracellular internalization analysis

To track the NPs and study the cellular uptake efficiencies, Fe_2_O_3_@TA-Pt NPs was firstly modified with OH-PEG-FITC and then cultured with 4T1 cells on confocal dish (100 µL Fe_2_O_3_@TA-Pt NPs, 5 mg/mL in medium) at 37 °C for 1 h. After washing with PBS (pH = 7.4) for twice, cells were stained with DAPI (20 µL) and LysoTracker Red DND-99 (5 µL) for 10 min, respectively. Subsequently, the cleaned cells were observed and recorded by confocal laser scanning microscope (CLSM 700, Zeiss, Germany).

### Study intracellular LPO generation

In this study, the fluorescent probe of BODIPY^581/591^-C11 was used to assess the intracellular LPO level. Briefly, 4T1 cells were pre-seeded in confocal dishes (5 × 10^5^ cells per dish) and incubated overnight at 37 °C. Then cells were treated with different samples (at an equivalent dosage of 100 µL, 5 mg/mL and RT dose was 1 Gy) and incubated for another 4 h. After that, cells were washed with PBS and cultured in the serum-free RMPI-1640 medium containing BODIPY^581/591^-C11 (5 µM) for 20 min. Subsequently, cells were washed and subjected to CLSM observation or flow cytometry analysis. The grouping is shown below: control group, Fe_2_O_3_ group (The cells were treated with Fe_2_O_3_ NPs), RT group (Only radiation therapy was given), Fe_2_O_3_ with RT group, Fe_2_O_3_@TA-Pt group and Fe_2_O_3_@TA-Pt with RT group. Specifically, the same was true of groups in other cell experiments.

### Pt-DNA adducts assay

The content of platinum coordinated with DNA were analyzed by ICP-MS (NexION 300 D, PerkinElmer Corporation, America). Firstly, 4T1 cells (5 × 10^5^) were seeded into 6-well plates for 24 h, and then cells pre-treated with NAC were incubated with 100 µL of Fe_2_O_3_@TA-Pt NPs (5 mg/mL in medium) and incubated at 37 °C for 20 min and 2 h, respectively. After that, cells were handled through washing and trypsinization to form a single cell suspension. The final genomic DNA was extracted and quantified by DNA extraction kit and platinum contents were determined by ICP-MS.

### DNA damage detection

4T1 cells were seeded in confocal dishes overnight, and then cells were treated with different methods (at an equivalent dosage of 100 µL, 5 mg/mL and RT dose was 1 Gy). After incubation for another 4 h, cells were washed with PBS (pH = 7.4) and cultured in the PBS containing anti-gamma H2A.X (Alexa Fluor 647) for 20 min. Additionally, the cytoskeleton and nucleus were labeled with DAPI and Phalloidin-iFluor 488 after cell fixation, respectively. And the final cell samples were subjected to CLSM observation.

### Intracellular ROS/hypoxia detection

4T1 cells were adopted to investigate intracellular ROS/hypoxia levels using an oxidative stress/hypoxia detection kit (Enzo Life Sciences). Cells were seeded in confocal dishes overnight at 37 °C. Then 100 µL of Fe_2_O_3_@TA-Pt NPs (5 mg/mL in medium) was added into the dishes and incubated for further 4 h. After that, cells were washed with PBS twice and treated with PBS containing hypoxia/oxidative stress detection mixture for 30 min. Washed with PBS for twice, Cells were analyzed under CLSM and flow cytometry (Cytomics FC 500 MCL, Beckman Coulter, America).

### Cellular GSH assay

After culturing at 37 °C for 24 h, 4T1 cells seeded in 6-well plates were treated with different samples. Then cells were harvested and washed with PBS for several times. Next, cells were lysed with Triton-X-100 lysis buffer and the GSH level was counted by GSH assay kit. The percentage content of GSH in cells was calculated based on the comparison to the GSH content of untreated cells.

### Western Blot analysis

4T1 cells were seeded in 6-well plate and treated with different handlings. Then the cell lysates were collected and analyzed by electrophoresis running on 14% denaturing polyacrylamide gels and the corresponding protein was transferred to the PVDF membrane. After blocking with 5% BSA, the membrane was incubated with primary antibodies (GPX4 and β-actin monoclonal antibodies) and secondary antibody (Goat anti-Rabbit IgG (H + L), HRP). The development mode was chemiluminescence (ECL) method.

### ***In vivo*** luminescence imaging

For diagnosing the tumor site and monitoring the NPs distribution in the body, the in vivo luminescence imaging of tumor was performed by IVIS Spectrum (Perkin Elmer, America). Briefly, Fe_2_O_3_@TA-Pt NPs were firstly modified with OH-PEG-Cy5 and then 4T1-tumor-bearing mice were intravenously injected with 200 µL of saline containing Fe_2_O_3_@TA-Pt NPs (5 mg/mL). The luminescence images were recorded at various time intervals, and after injection for 72 h, mice were sacrificed and the major organs were harvested and detected by in vivo imaging instrument.

### Biodistribution

200 µL of saline containing Fe_2_O_3_@TA-Pt NPs (5 mg/mL) were intravenously injected into 4T1-tumor-bearing mice for biodistribution analysis. Tumor bearing mice were sacrificed and major organs and tumor were harvested and weight at various time intervals (1, 2, 4, 8, 12, 24 h). By dissolving these organs in aqua regia, the Pt content was measured by an ICP-MS.

### Micropositron emission tomography (micro-PET imaging)

After post injection with 200 µL of Fe_2_O_3_@TA-Pt NPs (5 mg/mL in saline) for 12 h, 100 µL of saline suspension mixed with ^18^ F fluoromisonidazole ([^18^ F]MISO) (75 µCi/mouse) was intravenously injected into 4T1-tumor-bearing mice to evaluate the capacity of hypoxic degree within 72 h [34]. After injection with [^18^ F]MISO for an hour each time, PET scans were run on Inveon PET/CT system (Siemens, Malvern, PA, USA).

### ***In vivo*** antitumor study

4T1-tumor-bearing mice model was established by subcutaneous injection of 4T1 cells into Balb/c mice under the right front armpit. Once the volume of tumor reached an approximate size of 100 mm^3^, all the mice were randomly divided into six groups (10 mice in each group): control group, Fe_2_O_3_ group, RT group (Only radiation therapy was given), Fe_2_O_3_ with RT group, Fe_2_O_3_@TA-Pt group and Fe_2_O_3_@TA-Pt with RT group. These groups were intravenously injected with an equivalent dose of samples (200 µL, 5 mg/mL in saline), except control group (200 µL of saline) and RT group (200 µL of saline). After injection for 12 h, mice needed to be irradiated were treated with RT (2 Gy), and the same operation was repeated on the 7th day. To evaluate the effects and safety of treatment, the relative tumor volume, the body weight of mice and the survival of each mouse were recorded every two days. Furthermore, mice were sacrificed at different time intervals, and the tumors and primary organs (heart, liver, spleen, lung and kidney) were excised and collected for further analysis. Besides, the half of mice were used to study the survival situation in different groups till 50th day.

### Tumor tissue section analysis

In anti-tumor experiments, the tumors cell apoptosis and necrosis were measured by Hematoxylin and Eosin (H&E) staining and Terminal deoxynucleotidyl transferase (TdT) dUTP Nick-End Labeling (TUNEL) apoptosis staining. The proliferation of tumor cells was detected by ki67 staining. The positive vascular distribution in tumors was marked by CD31 staining. All the tissue sections were prepared by Nanjing KeyGEN BioTECH Company, and observed by fluorescence microscope (IX71, Olympus, Japan).

### Pathology analysis

After treating with Fe_2_O_3_@TA-Pt with RT, 4T1-tumor-bearing mice were sacrificed on first day, 7th day, 15th day and 30th day respectively to evaluate the chronic toxicity. The major organs (heart, liver, spleen, lung and kidney) were harvested and stored in 4% paraformaldehyde at room temperature. The sections were observed with hematoxylin and eosin (H&E) staining.

### Long-term cytotoxicity

Similar to pathology analysis, tumor-bearing mice were euthanized after 1, 7, 15, or 30 days, all the blood was compiled in heparin sodium anticoagulant vessels tube for hematology test. Briefly, alanine aminotransferase (ALT), aspartate aminotransferase (AST), total bilirubin level (TBIL) and total protein (TP) were used to indicate liver function. The levels of blood urea nitrogen (BUN) and creatinine (CRE) were evaluated to assess kidney function. Lymphocytes (LYM) levels were investigated to evaluate the immune response. Spleen function was evaluated by Platelet (PLT) production. Additionally, the inflammatory cytokines including TNF-α and IL-6 in the sera of mice were detected by Cytometric Bead Array (CBA) kit at the end of the 14 days treatment.

### Study on tumor recurrence and metastasis

After 14 days of treatment, 4T1-tumor-bearing mice treated with different rehabilitation strategy were euthanized and the corresponding lung were obtained. The surface metastatic nodules were counted and the internal status were analyzed by H&E and Ki67 staining. Furthermore, an additional 20 4T1-tumor-bearing mice were treated with synergistic treatment every 3 days until the tumor in the mouse was invisible. Then the tumor recurrence was checked every day over a 45-day observation period, and all the lungs were excised and collected for analysis of pulmonary metastasis.

### Statistical analysis

Quantitative data were analyzed by using Student’s t test by GraphPad Prism software (version 7.0) and the p-value of 0.05 or less was considered to be statistically significance.

## Results

### Characterization

**Fig. 1:**
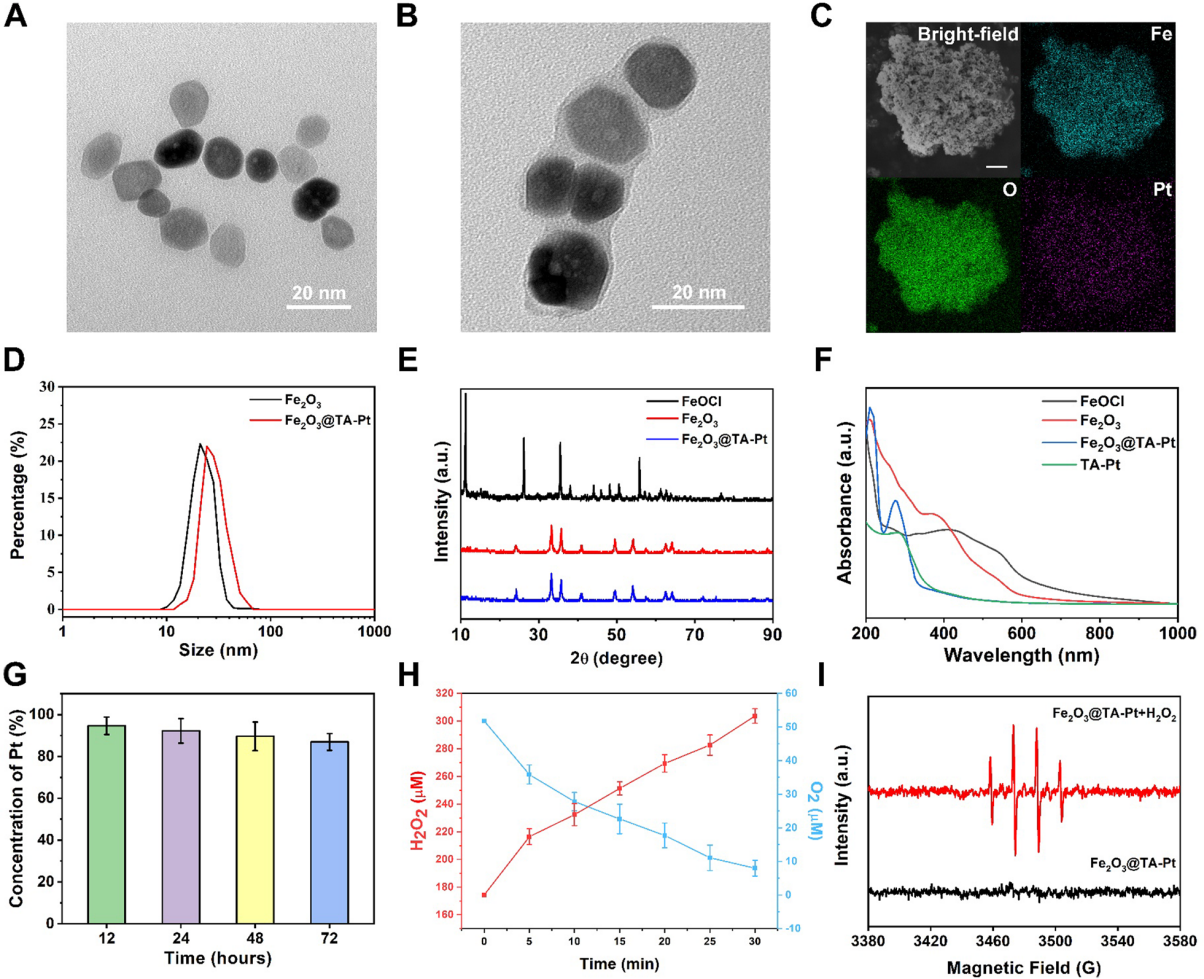
**A** TEM image of Fe_2_O_3_ NPs. **B** TEM image of Fe_2_O_3_@TA-Pt NPs. **C **EDS elemental mapping images of Fe_2_O_3_@TA-Pt NPs. Scale bar: 500 nm. **D** DLS results of Fe_2_O_3_ and Fe_2_O_3_@TA-Pt NPs. **E** Powder XRD patterns of Fe_2_O_3_ and Fe_2_O_3_@TA-Pt NPs. **F** UV-vis absorption spectra of FeOCl, Fe_2_O_3_, Fe_2_O_3_@TA-Pt NPs and TA-Pt complex. **G** The content of Pt in Fe_2_O_3_@TA-Pt NPs cultured with mouse serum. **H** Decomposition of H_2_O_2_ (red curve) and production of O_2_ (blue curve) by Fe_2_O_3_@TA-Pt NPs in aqueous solution (pH = 6.8) within 30 min. **I** EPR spectra of Fe_2_O_3_@TA-Pt NPs treated with or without H_2_O_2_ under acid condition

In this work, Fe_2_O_3_ NPs were obtained through a simple method, and their morphology and size can be controlled to meet different demand. Firstly, FeOCl NPs (Additional file 1: Fig. S1A) were synthesized as precursor to synthesize Fe_2_O_3_ NPs (Fig. 1A). These as-synthesized FeOCl NPs presented a mean particle size less than 20 nm with a petals shape, which was different from the conventional FeOCl nanosheet [35]. After treated with high temperature and pressure for 24 h, FeOCl NPs were completely converted to Fe_2_O_3_ NPs with a distinct outline and uniform particle size around 20 nm (Fig. 1B). In addition, the morphology and size of nanoparticles could be adjusted when the initial feeding amounts of FeOCl nanoparticles were changed (Additional file 1: Fig. S1B, C). The introduction of TA (Additional file 1: Fig. S2A) and Pt^2+^ resulted in a thin film on the surface of Fe_2_O_3_ NPs (Fig. 1B; Additional file 1: Fig. S3), probably due to the binding interaction between O (from TA) and Pt (Additional file 1:Fig. S2B). The energy-dispersive spectroscopy (EDS) mapping results analyzed by scanning electron microscopy (SEM) further confirmed the presence of Fe, O, and Pt in the final product of Fe_2_O_3_@TA-Pt NPs (Fig. 1C), and the mass ratio of Fe to Pt was approximately 155.36 obtained by inductively coupled plasma-mass spectrometer (ICP-MS) analysis (the loading rate of Pt was 12.86%). Fe_2_O_3_ NPs or Fe_2_O_3_@TA-Pt NPs could be stored in PBS (pH = 7.4) for up to four weeks without noticeable aggregation. Both of them were well-dispersed with narrow size distribution as revealed by dynamic light scattering (DLS) detection (Fig. 1D), which was consistent with the results of transmission electron microscopy (TEM). Figure 1E demonstrated the crystal structure of products obtained from X-ray diffraction (XRD). No characteristic peaks belonging to FeOCl NPs was observed in Fe_2_O_3_ NPs, confirming the complete conversion of FeOCl NPs to Fe_2_O_3_ NPs, and the coating of TA-Pt did not affect the crystal structure of Fe_2_O_3_ NPs. However, compared with pristine Fe_2_O_3_ NPs, a new characteristic peak was observed in the UV absorption curve of Fe_2_O_3_@TA-Pt NPs, probably owing to the existence of TA-Pt film (Fig. 1F). Furthermore, the change of Zeta-potential was observed (Additional file 1: Fig. S4A) after Fe_2_O_3_ NPs was coated with TA-Pt film, which also indicated the successful embellishment of TA-Pt film on the surface of Fe_2_O_3_ NPs.

To further verify the structure and composition of Fe_2_O_3_@TA-Pt NPs, we exploited X-ray photoelectron spectroscopy (XPS) detection to analyze the binding state of different elements on the surface of NPs. All typical peaks attributed to corresponding elements (C, O, Fe and Pt) were verified in Fe_2_O_3_@TA-Pt NPs (Additional file 1: Fig. S4B). Furthermore, the high-resolution signals of Pt 4f and Fe 3d in Fe_2_O_3_@TA-Pt NPs were presented in Additional file 1: Fig. S4C–F, indicating the presence of binding forces between Pt and O as well as the successful coating of TA-Pt film on the surface of Fe_2_O_3_ NPs. By further analyzing the iron with different valent states in Additional file 1: Fig. S4E, F, we found that the ratio of Fe^3+^ to Fe^2+^ on the surface of Fe_2_O_3_@TA-Pt NPs was approximately 1.56. And then, a significant decrease in the ratio of Fe^3+^ to Fe^2+^ (Fe^3+^/Fe^2+^=0.88) occurred after these NPs were treated with hydrogen peroxide under acidic condition for 1 h. The similar circumstances happened with platinum element, pure Pt^2+^ was converted to Pt^0^, Pt^2+^ and Pt^4+^ respectively under acidic condition and the rate was 1:1.41:1.77 (Additional file 1: Fig. S4C, D). The rapid increase of Fe^2+^ content ensured an adequate intracellular Fenton type reaction for efficient ferroptosis (Additional file 1: Fig. S5A), and the generation of oxygen in this process could improve the intra-tumoral oxygen pressure. It was reported that the acidic environment has a negative effect on the coordination capacity between TA and metal [36]. TA, as an ester substance, will be partially dissociated into gallic acid and glucose under acidic conditions (Additional file 1: Fig. S5B) [37]. Therefore, the TA-Pt film on the surface of Fe_2_O_3_ NPs would be impaired when Fe_2_O_3_@TA-Pt NPs were exposed to acidic condition. The broken TA-Pt film around Fe_2_O_3_ NPs has indeed been observed by TEM (Additional file 1: Fig. S6). Thus, the exposed Fe_2_O_3_ core could interact with hydrogen peroxide in tumor cells to produce ROS by Fenton type reaction, leading to ferroptosis-induced death of tumor cells and improve the therapeutic effect of CRT.

The stability of Fe_2_O_3_@TA-Pt NPs was also tested by incubating them in mouse serum and PBS with different pH values, respectively. Fe_2_O_3_@TA-Pt NPs were stable in mouse serum (Fig. 1G). Only a small amount of Pt was detected in the supernatant, and even after 72 h, less than 20% of Pt was released into the supernatant. After Fe_2_O_3_@TA-Pt NPs were incubated in PBS with acidic pH values, we noticed that higher amount of Pt was detected in PBS solution with the ascendant acidity, and around 70% of Pt was found in the PBS (pH 5.5) after 72 h (Additional file 1: Fig. S7A). Moreover, we compared the stability of Fe_2_O_3_ NPs and Fe_2_O_3_@TA-Pt NPs in mouse serum, and found that TA-Pt film improved the stability of Fe_2_O_3_ NPs (Additional file 1: Fig. S7B). These results indicated that Fe_2_O_3_@TA-Pt NPs presented a good stability in neutral condition and commendably responded to the acidic environment. Interestingly, the microenvironment of the tumor tissue is acidic and hypoxic with high level of H_2_O_2_ [38]. The etching of TA-Pt film in acidic tumor tissue would result in the exposure of iron cores, and H_2_O_2_ would convert Fe^3+^ to Fe^2+^ and generate O_2_ to relieve the hypoxia (Additional file 1:Fig. S5A). The decomposition of H_2_O_2_ and the production of O_2_ by incubating Fe_2_O_3_@TA-Pt NPs in acidic solution was confirmed in Fig. 1H. Finally, after being treated with acidic PBS and hydrogen peroxide, the hydroxyl radicals generated via Fenton Reaction were captured by 5,5-Dimethyl-1-pyrroline *N*-oxide (DMPO) (Fig. 1I). Hydroxyl radicals were continuously produced to hasten the effect of RT and ferroptosis until the iron source or hydrogen peroxide was exhausted, thereby leading to more tumor cell death including apoptosis-tolerant tumor cells.

### Cellular uptake and cytotoxicity

**Fig. 2:**
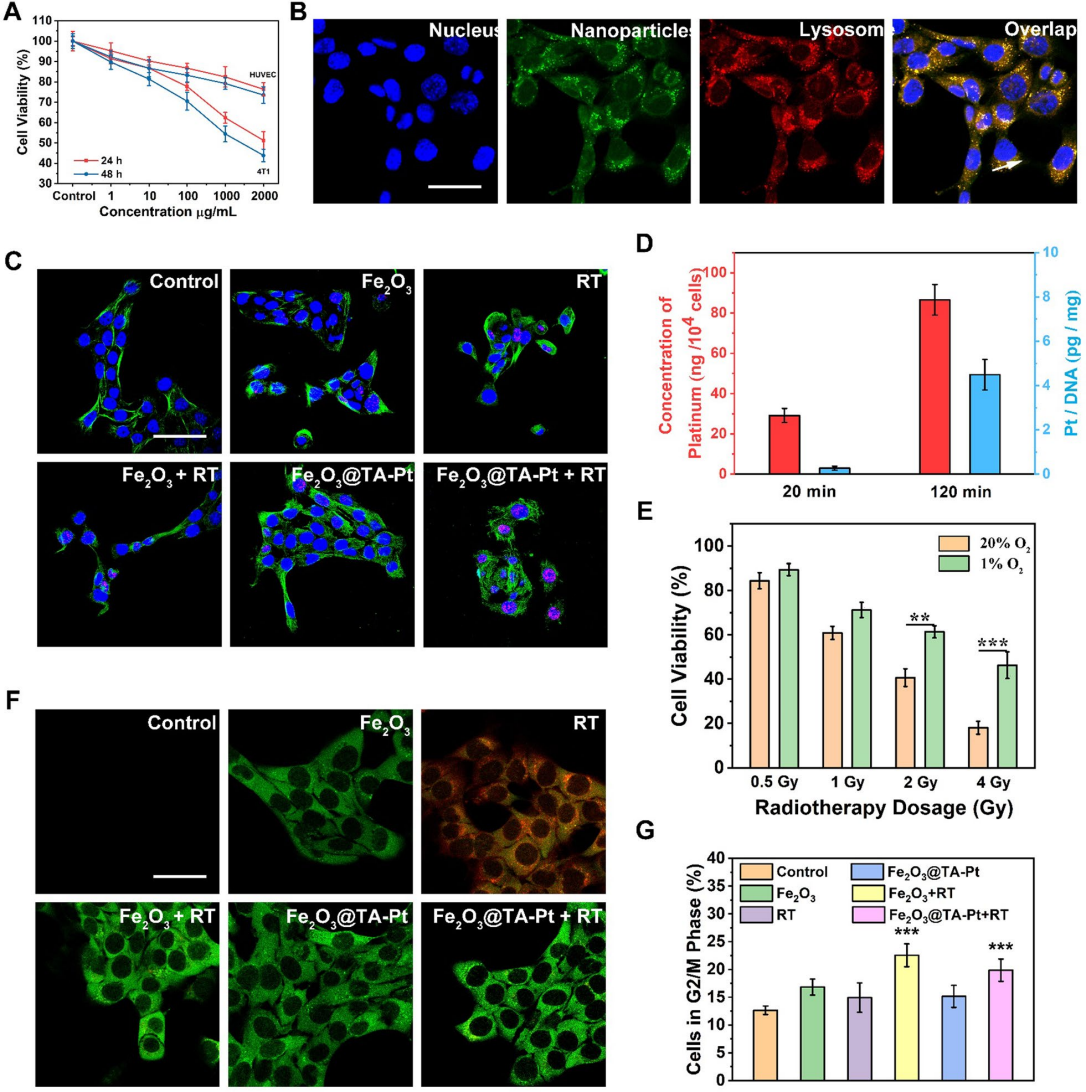
**A** In vitro viability of 4T1 cells and HUVEC incubated with Fe_2_O_3_@TA-Pt NPs for 24 or 48 h. **B** The endocytosis of Fe_2_O_3_@TA-Pt NPs. Scale bars: 10 μm. **C** The production of γ-H2A.X in 4T1 cells treated with different treatments (blue: DAPI, green: phalloidin, red: γ-H2A.X). Scale bars: 10 μm. **D** Platinum internalized in 4T1 cells (red) and corresponding Pt-DNA adducts (blue) after incubation with Fe_2_O_3_@TA-Pt NPs at different times (20 and 120 min). n = 3, ***p < 0.001. **E** In vitro viability of 4T1 cells treated with RT differed in dosage. n = 3, ***p < 0.001. **F** CLSM images of 4T1 cells subjected to different treatments, and ROS/hypoxia detection probes were used as indicators. Scale bars: 10 μm. **G** 4T1 cells in G2/M phase of cell cycle upon different treatments

The cytotoxicity caused by Fe_2_O_3_@TA-Pt NPs were examined by CCK-8 assay to verify whether these Fe_2_O_3_@TA-Pt NPs could be used in the tumor therapy (Fig. 2A). For normal cells, even when the cells were challenged by Fe_2_O_3_@TA-Pt NPs with concentration as high as 2000 µg/mL, more than 80% cells were alive. Inversely, nearly 60% of the tumor cells were killed after 48 h incubation, indicating a low cytotoxicity against normal cells and excellent anti-tumor activity of Fe_2_O_3_@TA-Pt NPs. Figure 2B and Additional file 1: Fig. S8 illustrated the cellular uptake results of Fe_2_O_3_@TA-Pt NPs by tumor cells. Majority of Fe_2_O_3_@TA-Pt NPs (green) were internalized by 4T1 cells within 1 h, overlapping with lysosomes (red), in consistent with the endocytosed path through the endo-lysosome network. The co-localization analysis of green and red color depicted by the white arrow in Fig. 2B also confirmed that these Fe_2_O_3_@TA-Pt NPs entered cells through endocytosis (Additional file 1: Fig. S8).

### Chemotherapy and RT study

In this study, TA-Pt film could not only improve the hydrophilicity and stability of Fe_2_O_3_ NPs, but also interact with DNA to prevent tumor cell proliferation like platinum chemotherapy agents did. As shown in Additional file 1: Figs. S9, S10 and Fig. 2C, ROS in tumor cells was firstly quenched by N-acetylcysteine (NAC) to eliminate relevant interference. Then DNA double-strand damages were detected by measuring γ-H2A.X protein content (red dots). We noticed that the DNA was damaged by Pt complex produced from the dissociation of TA-Pt film (Additional file 1: Fig. S9), and the radiotherapy (RT) treatment alone also caused damage to DNA (Additional file 1: Fig. S10; Fig. 2C). The most significant intensity of red color was seen in the cells treated by Fe_2_O_3_@TA-Pt NPs plus RT, demonstrating the maximal damage to the DNA segment due to the synergistic effect of RT and Pt complex. To further confirm that Pt complex could cause DNA damage, the genomic DNA was stripped out and collected to analyze the cross-linking capacity between Pt and DNA through testing the content of Pt via ICP-MS (Fig. 2D). Obviously, the amount of Pt in cells increased upon the extension of incubation time, indicating more Fe_2_O_3_@TA-Pt NPs inside cells. We further noticed that the amount of Pt embedded with DNA was also enlarged with the extension of incubation time, which mean that more Pt would bind with DNA to prevent tumor cells proliferation.

RT could not only produce ROS to induce apoptosis, but also directly destroy DNA and proteins in tumor cells to induce necrosis. However, the effect of RT in cancer treatment was greatly restricted due to the serious hypoxia in tumor tissue [39]. In our system, H_2_O_2_ would be converted to O_2_ to relieve the hypoxia with the help of Fe_2_O_3_@TA-Pt NPs, which would confer an enhanced anti-tumor effect on RT. As seen in Fig. 2E, to evaluate the dependence of RT on oxygen, 4T1 cells were irradiated with a gradual increasing dose of RT after being incubated under normoxia (20% O_2_) or hypoxia (1% O_2_) conditions, respectively. Notably, the lack of oxygen suppressed the killing effect of RT, especially in high doses RT. The application of RT could cause the serious hypoxia while inducing less amount of ROS in tumor cells (Fig. 2F; Additional file 1: Fig. S11). The presence of Fe_2_O_3_@TA-Pt NPs would greatly relieve the hypoxic degree in tumor cell due to the generation of O_2_, consequently leading to a high amount of ROS. The relief of hypoxia and the enrichment of O_2_ both enhanced the radio-sensitivity of tumor cells, which would benefit the RT effect. In addition, the improvement of intracellular oxygen was also conducive to a transition to G2/M phase of cell cycle, during which the RT sensitivity of cells was the highest, leading to enhanced radiotherapy effect (Fig. 2G). The therapeutic effect of RT could also be improved by the secondary radiation effect (Compton scattering and Auger effect) of metal NPs. By continuously incubating cells with NAC before RT to avoid the impact from ROS, the death rate of cells treated with Fe_2_O_3_ NPs was still higher than that of the untreated tumor cells (Additional file 1: Fig. S12), which can be partly explained by the secondary radiation generated by Fe_2_O_3_ NPs. Therefore, the effect of RT was effectively improved, even with a low dose, greatly avoiding additional side effects.

### Ferroptosis study

**Fig. 3:**
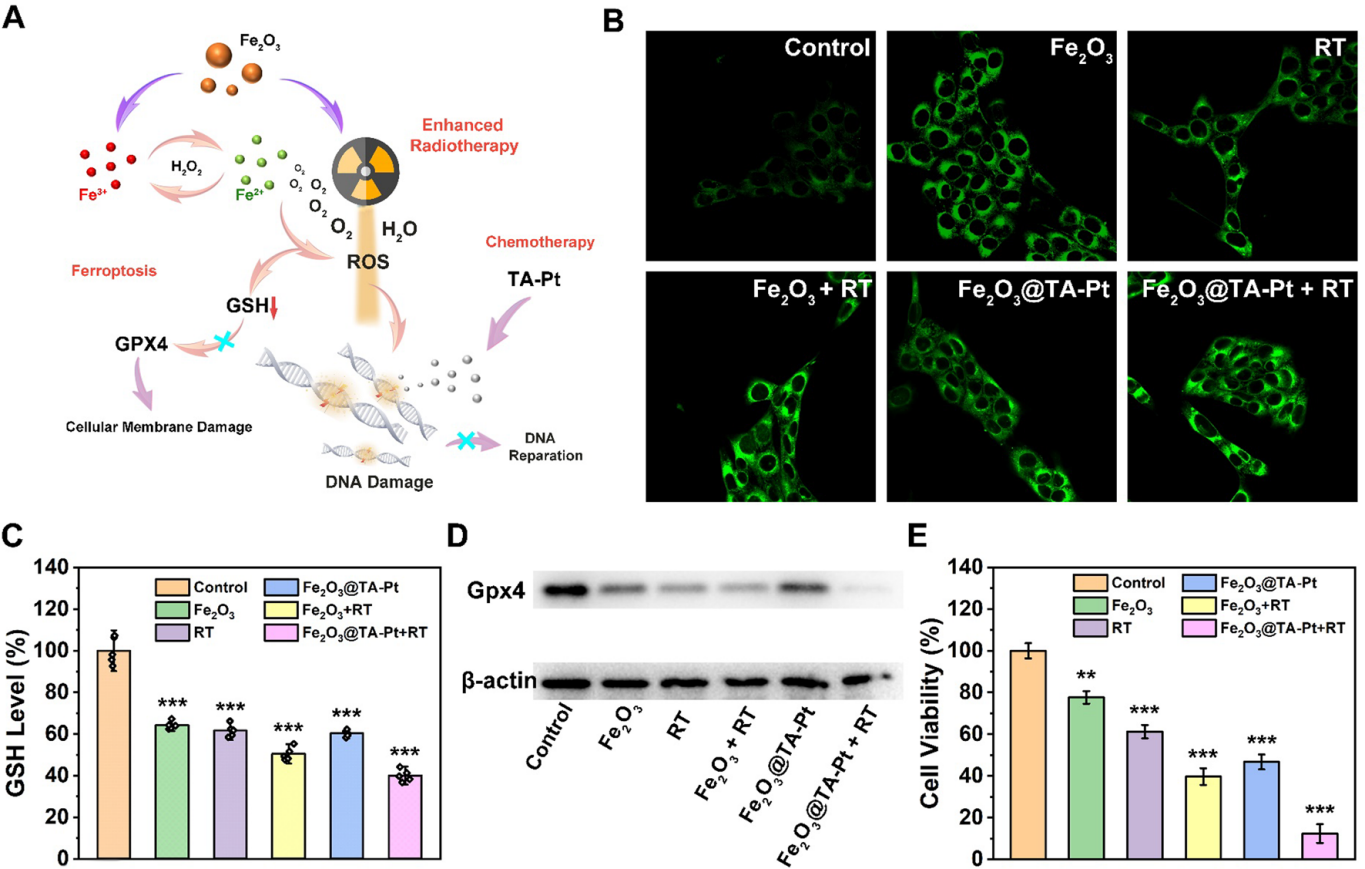
**A** Cellular factors involved in Fe_2_O_3_@TA-Pt-mediated tumor therapy. **B** BODIPY^581/591^-C11 staining CLSM images after the treatment with different types of samples. Scale bar: 10 μm. **C** Intracellular GSH level of 4T1 cells after treated with different treatments. n = 3, ***p < 0.001. **D** Western blot analysis of GPX4 expression in 4T1 cells. **E** In vitro viability of 4T1 cells treated with different treatments. n = 7, **p < 0.01, ***p < 0.001

Different from apoptosis and necrosis, ferroptosis bypasses these related pathways and induces the peroxidation of cell membrane [40]. Especially for apoptosis-resistant tumor cells, ferroptosis can eliminate them well, thus preventing tumor metastasis and recurrence. In addition, ROS was continuously produced in this process, which could further damage the DNA, preventing tumor cells from repairing itself and enhancing the effects of RT. Under the synergistic action of ROS and platinum-based chemotherapy drugs, tumor cells could be eliminated to the greatest extent (Fig. 3A).

To monitor the changes of cell membrane status induced by ferroptosis, BODIPY^581/591^-C11 probe was employed to detect the LPO levels in cell membrane (Fig. 3B; Additional file 1: S13A). Compared with control group, different levels of LPO were observed among other groups, and the highest level of LPO was found in Fe_2_O_3_@TA-Pt NPs plus RT group, indicating that severe cell membrane peroxidation was triggered by ferroptosis path, especially in the combination of Fe_2_O_3_@TA-Pt NPs and RT. After receiving RT, cells pre-incubated with Fe_2_O_3_ NPs or Fe_2_O_3_@TA-Pt NPs produced significantly higher levels of ROS than those of cells without the treatment of NPs, due to the increased oxygen pressure in tumor cells caused by Fe_2_O_3_ NPs or Fe_2_O_3_@TA-Pt NPs. More importantly, under the same condition, cells treated with RT presented more severe cell membrane peroxidation, illustrating that ROS produced by RT could also destroy cell membranes (Additional file 1: Fig. S13B).

Lipid repair enzyme-glutathione peroxidase 4 (GPX4), acting as an important antioxidant of cell membrane, was mainly produced by glutathione (GSH), which could be consumed by ROS. As seen in Fig. 3C, the GSH level significantly declined after the combined treatment of Fe_2_O_3_@TA-Pt NPs and RT and the content of GSH in the cells treated with RT was lower than that in the cells without RT, indicating the high level of ROS generated by RT. The content of GPX4 protein was measured by western blot (Fig. 3D; Additional file 1: Fig. S14). As expected, the levels of GPX4 declined to some extent among all samples, which was also consistent with the results of GSH levels. In addition, the change of mitochondrial character is another important characteristic of ferroptosis [41]. Tumor cells treated with different samples were fixed in 2.5% glutaraldehyde and collected for TEM observation (Additional file 1: Fig. S15). For control, the mitochondria remained in a normal state with clear internal structures, while shrinking mitochondria, atrophy, damage of the inner ridge, and increased membrane density were observed in other cells, illustrating that tumor cells were suffering from ferroptosis. In addition, caspase-3-dependent apoptotic pathways were activated by these treatments, especially in the Fe_2_O_3_@TA-Pt NPs plus RT group (Additional file 1: Fig. S16), indicating that our nanoplatforms could also activate the caspase-dependent apoptosis paths for tumor therapy. Finally, we tested the cell viability of 4T1 cells receiving different treatments and noticed that almost 90% of the tumor cells were killed by Fe_2_O_3_@TA-Pt NPs plus RT (Fig. 3E), unambiguously revealing a broad prospect of anti-tumor effect.

### ***In vivo*** distribution profiles

**Fig. 4:**
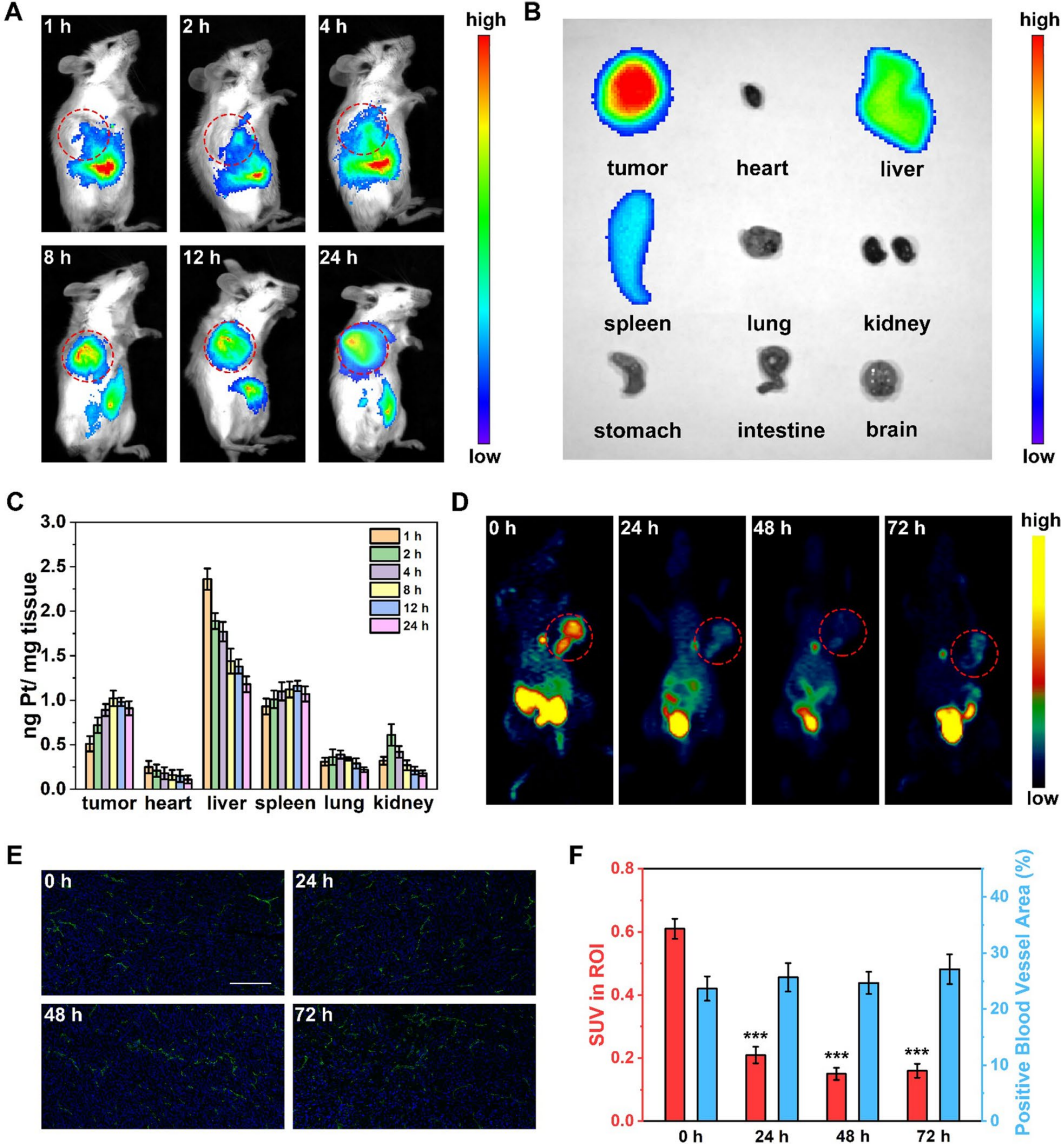
**A** In vivo fluorescence imaging of mice received intravenous injection of Cy5.5-labeled Fe_2_O_3_@TA-Pt. These images were collected at 1, 2, 4, 8, 12, and 24 h post-injection. **B** The fluorescence images of organs harvested at 72 h post-injection. **C** In vivo biodistribution of Pt at different time periods (1, 2, 4, 8, 12 and 24 h). **D** PET images of mice with different treatments (the red circles indicated tumors, different colors represented different levels of [^18^ F]MISO uptake), the PET was conducted 1 h after the injection of [^18^ F]MISO. **E** Immunofluorescence images showing the distribution of CD31-positive blood vessels (green) and nuclei (blue) in tumor tissue at different time points. Scale bar: 100 μm. **F** Standardized uptake values (SUVs) of [^18^ F]MISO in tumor obtained from PET images (left panel) and relative blood vessel area as shown in **E** (right panel). n = 3, ***p < 0.001

Accurately tracking the distribution of NPs in the body is another important factor to evaluate their *in vivo* safety and reliability [42]. As shown in Fig. 4A, most of the Fe_2_O_3_@TA-Pt NPs were enriched in mouse liver within the initial 4 h, and gradually accumulated in the tumor tissue with the extension of time. After 72 h, the accumulation of Fe_2_O_3_@TA-Pt NPs occurred mainly in mononuclear phagocyte system-associated organs (liver and spleen) and tumor (Fig. 4B). These results were further supported by subsequent Pt content analysis via ICP-MS (Fig. 4C), illustrating that Fe_2_O_3_@TA-Pt NPs could escape from phagocytic cells or the reticuloendothelial system (RES) and get stuck in tumors.

### Positron emission tomography (PET) imaging

Reacted with H_2_O_2_ in tumors to generate oxygen to alleviate hypoxia, Fe_2_O_3_@TA-Pt NPs could enhance the oxygen-dependent treatment. We employed hypoxia-sensitive [^18^ F]MISO probe to monitor the hypoxia at 4T1-tumor-bearing mice, and noticed that hypoxia in the tumor was rapidly relieved and did not deteriorate for a long time (Fig. 4D, F). In addition, no significant angiogenesis occurred within the duration of treatment (Fig. 4E, F). The factor responsible for reoxygenation was due to the internal oxygen supply from the decomposition of H_2_O_2_, which was consistent with the abovementioned results (Fig. 1H; Additional file 1: S5A). Therefore, Fe_2_O_3_@TA-Pt NPs could accumulate in tumor tissue by the enhanced permeability and retention effect, and relieve the hypoxia to provide a powerful support to tumor therapy.

### In vivo antitumor effect

**Fig. 5:**
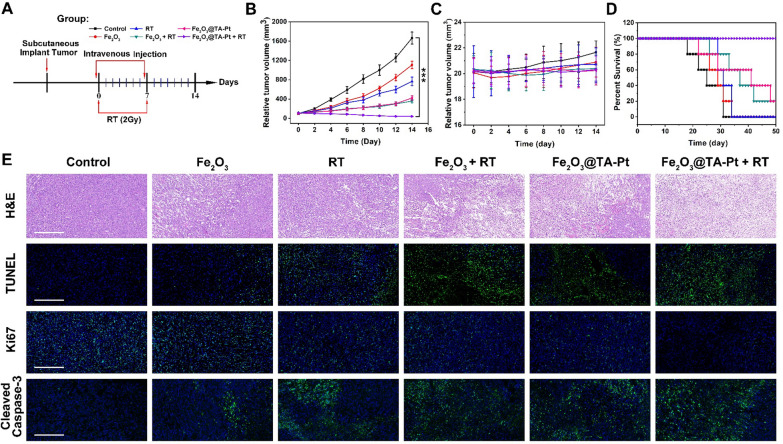
**A** The preset grouping and program for anti-tumor experiment. **B** Relative tumor volume and **C** body weight profiles of 4T1-tumor-bearing mice receiving different treatments grouped according to **A**. n = 5, ***p < 0.001. **D** Survival profiles of mice within a period of 14 days post treatment (Grouping was as shown in **A**). **E** Images of tumor tissue section stained with H&E, TUNEL, Ki67 and Cleaved Caspase-3 in different groups. Scale bar: 100 μm

To systematically investigate the anti-cancer ability of Fe_2_O_3_@TA-Pt NPs, 4T1-tumor-bearing mice were intravenously injected with different samples following the preset grouping and program as depicted in Fig. 5A. According to the schedule, mice were intravenously injected with 200 µL of saline containing the same dose of NPs (5 mg/mL) 12 h before RT and the same procedure was repeated on the 7th day to consolidate the curative effect. In this case, tumor volumes were recorded every two days (Fig. 5B). The tumor volume of mice only receiving the injection of saline approached 1000 mm^3^ within 10 days. By contrast, mice in other groups appeared varying degrees of inhibition to the progressive tumor, especially in Fe_2_O_3_@TA-Pt plus RT group, showing a retarded tumor outgrowth with the most significant tumor regress. In addition, we recorded the body weight of tested mice every two days (Fig. 5C), and found that there was no dramatic weight loss for these mice, demonstrating less acute toxicity to these animals. Figure 5D showed the survival profiles of mice in each group. Compared to the control group, the group of Fe_2_O_3_@TA-Pt NPs plus RT effectively extended the life of the mice. Even after 50 days, all the mice in this group were still alive.

At the end of the 14-day treatment, we harvested the tumor tissues and stained the sections with H&E to evaluate the histological change of tumors (Fig. 5E). Compared with the control group with dense tissue structure, atrophy, apoptosis or necrosis were discovered in these treated groups, and the most serious tissue damage was witnessed in the Fe_2_O_3_@TA-Pt NPs plus RT group. The assessment by TUNEL assay further supported the above observation, from which abundant apoptotic cells (green) was filled in the whole tumor tissue. The signal protein of Ki67, indicating the proliferation and differentiation of tumor cells, performed a sharp decline in Fe_2_O_3_@TA-Pt NPs plus RT group, disclosing the attenuated activity for tumor cells. Additionally, the caspase-3-dependent apoptotic pathways were activated by these rehabilitation strategies, in consistent with cell level analysis data, and a large amount of cleaved caspase-3 (green) were expressed in tumor cells, demonstrating the necessity of ferroptosis in the treatment of apoptotic-tolerant tumor cells. All these observations manifested that the combinational treatment not only eradicated the cancer cells, but more importantly, remodeled the tumor niche towards someone disfavoring the growth of cancer cells.

### Pathological analysis and hematology assay

To evaluate the safety and reliability of combinational therapy, we harvested the blood and major organs from the 4T1-tumor-bearing mice treated with Fe_2_O_3_@TA-Pt NPs under a low doses RT at different time points. Although some small fluctuations of various indicators happened in hematology assays after treatment, every indicator went back to normal status, including the immune function, liver function, spleen function and renal function (Additional file 1: Fig. S17). In addition, we did not notice any significant necrosis or apoptosis in the section of the H&E assays from vulnerable tissues (Additional file 1: Fig. S18). Therefore, Fe_2_O_3_@TA-Pt NPs plus a low dose RT held the promise to serve as a biocompatible candidate for conquering cancer.

### Inhibition of tumor recurrence and metastasis

**Fig. 6:**
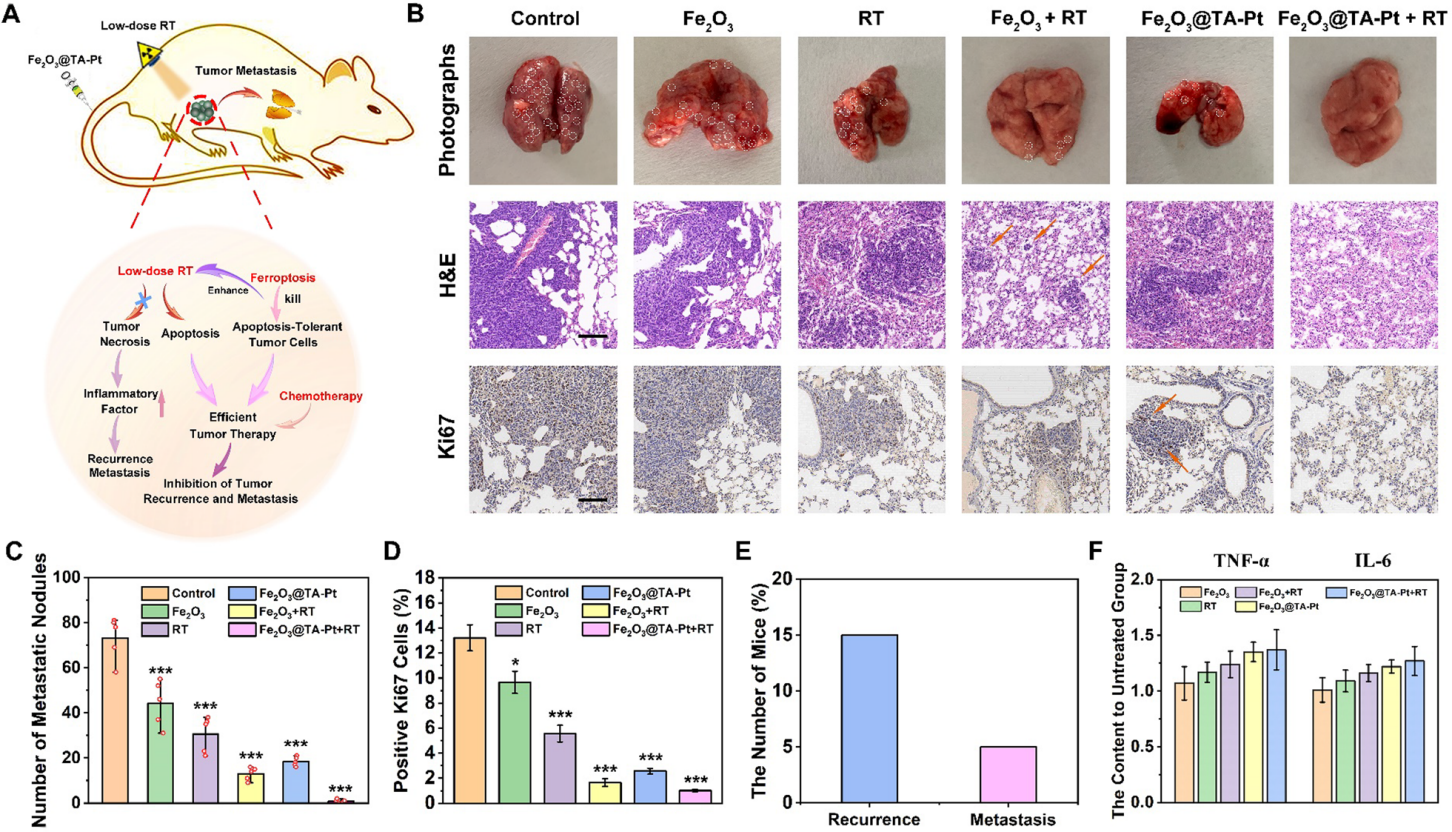
**A** Schematic illustration of Fe_2_O_3_@TA-Pt NPs to inhibit tumor recurrence and metastasis. **B** The photographs of lung and relevant images of lung section stained with H&E and Ki67 in different groups. Scale bar: 100 μm, (the red circles represented metastatic nodules and Ki67 positive cells). **C** The number of metastatic nodules in lung from different groups. n = 10, ***p < 0.001. **D** Positive Ki67 cells counted from Ki67 staining section by using ImageJ software. n = 10, n = 3, *p < 0.05, ***p < 0.001. **E** Proportion of mice with recurrence and lung metastasis after combination treatment, n = 20. **F** The content of TNF-α and IL-6 in sera of mice after treatment

Tumor recurrence and metastasis as commonplace in tumor therapy, are mainly attributed to the incomplete depletion of tumor, including the low therapeutic efficiency, the inability of removing apoptotic-tolerant tumor cells and the side effect from vast tumor necrosis [43]. In this work, the combination of Fe_2_O_3_@TA-Pt NPs and RT can not only efficiently kill hypoxic tumor cells, but also eradiate the apoptosis-resistant tumor cells to ensure less tumor recurrence and metastasis (Fig. 6A). As observed in Fig. 6B and C 4T1 cells rapidly metastasized from the primary tumor to lung in control group, while the tumor metastasis was inhibited with varying degrees in other groups, and almost no metastatic nodules was observed in the Fe_2_O_3_@TA-Pt NPs plus RT group. These sections of lung tissue were also observed by H&E staining and the dense tissue structure of metastatic nodules were more clearly identified among all the lungs except that from Fe_2_O_3_@TA-Pt NPs plus RT group. In addition, we also employed Ki67 to analyze the proliferative activity of tumor cells in metastatic nodules (Fig. 6B, D). A large number of tumor cells with positive Ki67 were observed in untreated group, suggesting a continually deterioration in the metastatic tumor. However, the similar situation did not occur in mice treated with Fe_2_O_3_@TA-Pt NPs under a low dose RT, indicating that our nanoplatforms possessed a superior antineoplastic activity and could efficiently inhibit tumor recurrence and metastasis.

4T1-tumor-bearing mice were also treated with Fe_2_O_3_@TA-Pt NPs under a low dose RT every three days until no tumor could be seen. Then the tumor recurrences were recorded daily in the following 45 days. After harvesting the lungs from these mice, we found that merely 15% of mice suffered from tumor recurrences and 5% of mice experienced pulmonary metastasis (Fig. 6E). The content of TNF-α and IL-6 were tested in the sera of mice at the end of the 14-day treatment and shown in Fig. 6F. The levels of these two factors increased mildly, suggesting that RT with a low dose induced apoptosis without severe inflammation, thus avoiding the tumor recurrence and metastasis. Overall, these results indicated that the combinational therapy strategy in this study presented promising prospects for excellent CRT and the inhibition of tumor recurrence and metastasis.

## Conclusions

In summary, TA-Pt film was covered on the surface of Fe_2_O_3_ NPs to enhance their stability, and then displayed a chemotherapeutic effect to interrupt cell growth after responding to acidic tumor microenvironment. Followed by this, the presence of H_2_O_2_ in tumors vulnerated the exposed Fe_2_O_3_ core to produce oxygen and ROS to induce ferroptosis in tumor cells. Moreover, the low dose of RT benefited by these generated oxygens efficiently increased the intracellular ROS content for enhanced CRT, and reduced the potential risk of tumor recurrence and metastasis without causing other side effects. Encouraged by the long-term protection from our nanoplatform as well as its low toxicity, we believe it could play an active role against tumor and tumor associated recurrence and metastasis in clinic.

## Supplementary Information

**Additional file 1: Figure S1. **A) TEM image of FeOCl NPs. B) TEM image of FeOCl NPs (One-tenth of ingredients). C) TEM image of Fe_2_O_3_NPs (One-tenth of ingredients). **Figure S2**. A) Structural of TA. B) Schematicillustration of the binding between Pt^2+^and TA. **Figure S3**. SEM of Fe_2_O_3_@TA-PtNPs. **Figure S4**. A) Zeta-potential of Fe_2_O_3_and Fe_2_O_3_@TA-Pt NPs as measured in PBS (pH=6.8, 37 ^o^C)B) XPS spectrum of Fe_2_O_3_@TA-Pt NPs. Highresolution Pt 4f spectra of Fe_2_O_3_@TA-Pt NPs C) before and D) after treating with H_2_O_2_ under acidic conditions(pH=6.8) for 1 h. High resolution Fe 3d spectra of Fe_2_O_3_@TA-Pt NPs E) before and F) after treating with H_2_O_2_ underacidic conditions (pH=6.8) for 1 h. **Figure S5**. A) Schematic illustration ofhydroxyl radical generation. B) Schematic illustrationof TA hydrolyzes under acidic conditions. **Figure S6**. TEM image of Fe_2_O_3_@TA-Pttreated with acidic PBS (pH=6.8) for 24 h. **Figure S7**. A) The content of Pt in Fe_2_O_3_@TA-Pt NPs cultured with different PBS (pH=7.4, 6.8, 5.5). B) The content of Fe in Fe_2_O_3_NPs and Fe_2_O_3_@TA-Pt NPs cultured with mouse serum for 72 h. **Figure S8**. Fluorescence intensity profiles of Fe_2_O_3_@TA-Pt NPs and lysosome were measured using Fiji(Image J) and shown as a function of distance. **Figure S9**. The production of γ-H2A.X in 4T1cells pre-incubated with NAC to analyze DNA damage caused by TA-Pt film. Scalebars: 10 μm. **Figure S10**. The independent CLSM images of 4T1cells for testing γ-H2A.X with different treatments (blue: DAPI, green:phalloidin, red: γ-H2A.X). Scale bar: 10 μm. **Figure S11**. The independent CLSM images of 4T1cells for ROS/hypoxia detection. Scalebar: 10μm. **Figure S12**. *In vitro* viability of 4T1 cells pre-incubated with NACto analyze the. **Figure S13**. A) Flow cytometry of LPO levelsafter cells were treated with different types of treatments as displayed. B)The fluorescence intensities of ROS generated in 4T1 cells during differenttypes of treatments and measured by flow cytometry. **Figure S14**. The gray ratio of western blotanalysis (GPX4) to the control group. **Figure S15**. Cells morphology with Fe_2_O_3_@TA-Pt-mediatedferroptosis after incubated with NPs or irradiation for 24h. **Figure S16**. Cleaved Caspase-3 analysis of 4T1 cells subjected to different treatments. Scale bars:10 μm. **Figure S17**. The hematology assay of 4T1-tumor-bearing mice with Fe_2_O_3_@TA-Pt under radiotherapy at days 1, 7, 15, and 30. **Figure S18**. The long-term toxicity of Fe_2_O_3_@TA-Ptin heart, liver, spleen, lung, and kidney at days 1, 7, 15, and 30 post intravenous injection of Fe_2_O_3_@TA-Pt NPs using H&Estaining. Scale bars: 100 μm.

## Data Availability

All data generated or analyzed during this study are included in this published article.

## Abbreviations

CRT: Concurrent chemoradiotherapy
ROS: Reactive oxygen species
TA-Pt: Tannic acid (TA) and Pt^2+^
RT: Radiotherapy
GSH: Glutathione
GPX4: Glutathione peroxidase 4
LPO: Lipid hydroperoxides
4T1: Mouse Breast cancer
HUVEC: Human umbilical vein edothelial cells
[^18^F]MISO: ^18^F fluoromisonidazole
H&E: Hematoxylin and eosin
TUNEL: Terminal deoxynucleotidyl transferase (TdT) dUTP nick-end labeling
