# Correction to: Platinum prodrug nanoparticles inhibiting tumor recurrence and metastasis by concurrent chemoradiotherapy

**DOI:** 10.1186/s12951-022-01418-5

**Published:** 2022-05-19

**Authors:** Wei Jiang, Lulu Wei, Bing Chen, Xingyu Luo, Peipei Xu, Jianfeng Cai, Yong Hu

**Affiliations:** 1 Department of Hematology, Nanjing Drum Tower Hospital, Nanjing University, Nanjing, 210093 China; 2 Institute of Materials Engineering, College of Engineering and Applied Sciences, Nanjing University, Jiangsu, 210093 China; 3 Department of Chemistry, University of South Florida, 4202 E. Fowler Ave, Tampa, FL 33620 USA

## Correction to: Journal of Nanobiotechnology (2022) 20:129 https://doi.org/10.1186/s12951-022–01322-y

Following publication of the original article [1], the author reported that the corresponding author symbols were omitted from the author group. Dr. Jianfeng Cai and Peipei Xu have been added to the author group and are presented correctly in this correction article. There are 3 corresponding authors, including Ms. Yong Hu (hvyong@nju.edu.cn), Jianfeng Cai (jianfengcai@usf.edu) and Peipei Xu (xu_peipei0618@163.com).

The original article [1] has been corrected.

## References

[1] Jiang W, Wei L, Chen B, Luo X, Xu P, Cai J, Hu Y (2022). Platinum prodrug nanoparticles inhibiting tumor recurrence and metastasis by concurrent chemoradiotherapy. J Nanobiotechnol.

