# Supplementary material for: Platinum prodrug nanoparticles inhibiting tumor recurrence and metastasis by concurrent chemoradiotherapy
Source: J Nanobiotechnology. 2022 Mar 12;20:129. doi: 10.1186/s12951-022-01322-y (PMC8917711; doi:10.1186/s12951-022-01322-y)
Supplement: Supplementary file 1 — Additional file 1: Figure S1. A) TEM image of FeOCl NPs. B) TEM image of FeOCl NPs (One-tenth of ingredients). C) TEM image of Fe2O3NPs (One-tenth of ingredients). Figure S2. A) Structural of TA. B) Schematicillustration of the binding between Pt2+and TA. Figure S3. SEM of Fe2O3@TA-PtNPs. Figure S4. A) Zeta-potential of Fe2O3and Fe2O3@TA-Pt NPs as measured in PBS (pH=6.8, 37 oC)B) XPS spectrum of Fe2O3@TA-Pt NPs. Highresolution Pt 4f spectra of Fe2O3@TA-Pt NPs C) before and D) after treating with H2O2 under acidic conditions(pH=6.8) for 1 h. High resolution Fe 3d spectra of Fe2O3@TA-Pt NPs E) before and F) after treating with H2O2 underacidic conditions (pH=6.8) for 1 h. Figure S5. A) Schematic illustration ofhydroxyl radical generation. B) Schematic illustrationof TA hydrolyzes under acidic conditions. Figure S6. TEM image of Fe2O3@TA-Pttreated with acidic PBS (pH=6.8) for 24 h. Figure S7. A) The content of Pt in Fe2O3@TA-Pt NPs cultured with different PBS (pH=7.4, 6.8, 5.5). B) The content of Fe in Fe2O3NPs and Fe2O3@TA-Pt NPs cultured with mouse serum for 72 h. Figure S8. Fluorescence intensity profiles of Fe2O3@TA-Pt NPs and lysosome were measured using Fiji(Image J) and shown as a function of distance. Figure S9. The production of γ-H2A.X in 4T1cells pre-incubated with NAC to analyze DNA damage caused by TA-Pt film. Scalebars: 10 μm. Figure S10. The independent CLSM images of 4T1cells for testing γ-H2A.X with different treatments (blue: DAPI, green:phalloidin, red: γ-H2A.X). Scale bar: 10 μm. Figure S11. The independent CLSM images of 4T1cells for ROS/hypoxia detection. Scalebar: 10μm. Figure S12. In vitro viability of 4T1 cells pre-incubated with NACto analyze the. Figure S13. A) Flow cytometry of LPO levelsafter cells were treated with different types of treatments as displayed. B)The fluorescence intensities of ROS generated in 4T1 cells during differenttypes of treatments and measured by flow cytometry. Figure S14. The gray ratio of western blotanalysis (GP [file 12951_2022_1322_MOESM1_ESM.docx]

Additional file 1


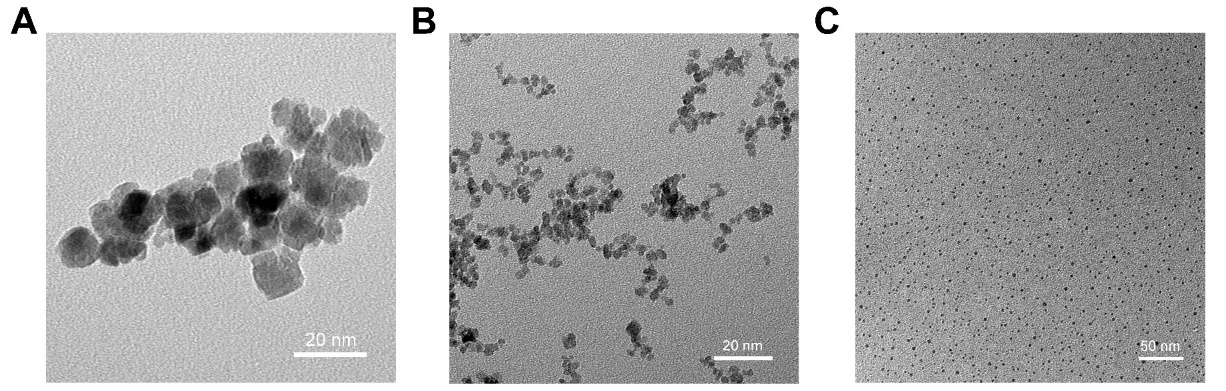


Figure S1. A) TEM image of FeOCl NPs. B) TEM image of FeOCl NPs (One-tenth of ingredients). C) TEM image of Fe_2_O_3_ NPs (One-tenth of ingredients).


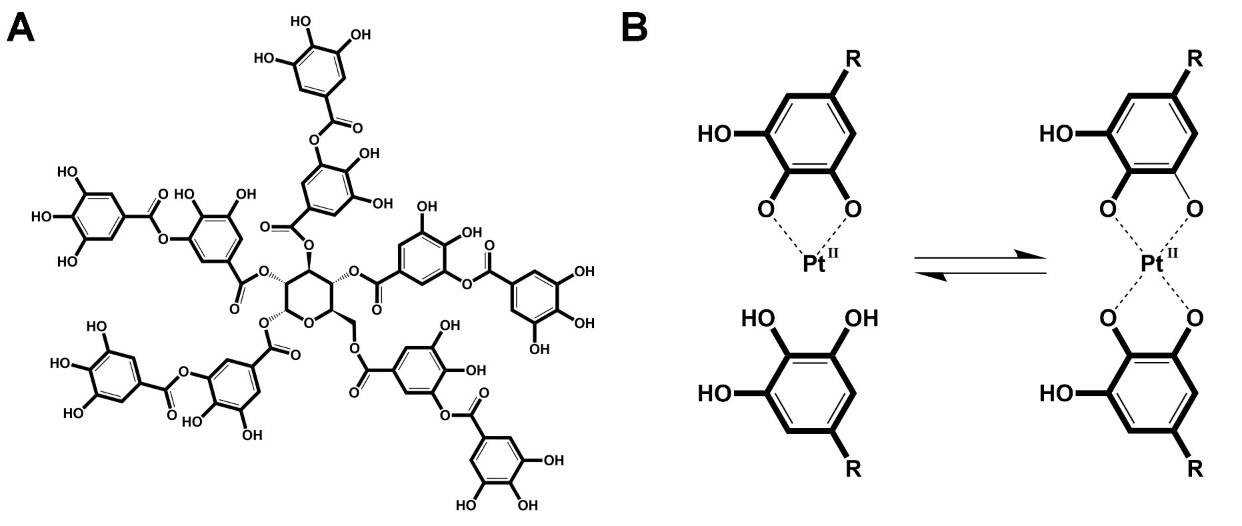


Figure S2. A) Structural of TA. B) Schematic illustration of the binding between Pt^2+^ and TA.


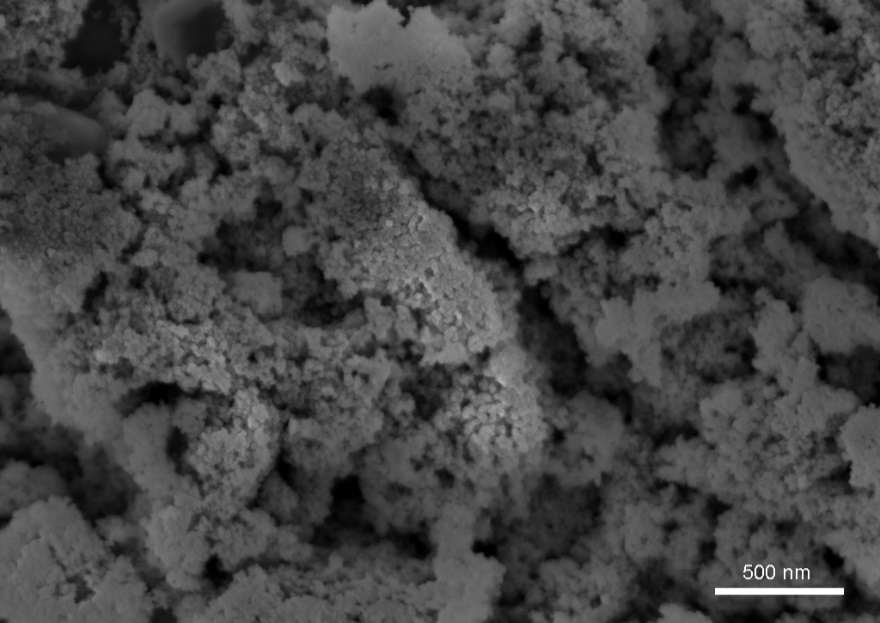


Figure S3. SEM of Fe_2_O_3_@TA-Pt NPs.


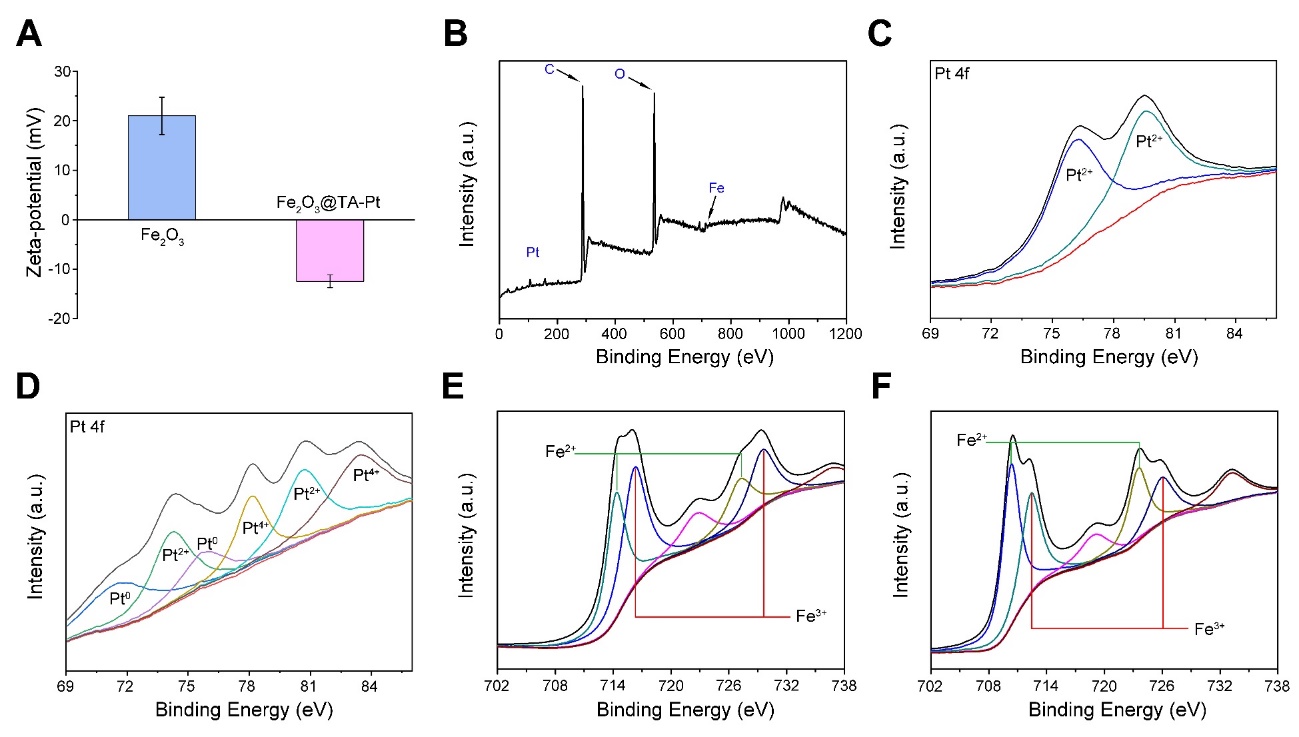


Figure S4 A) Zeta-potential of Fe_2_O_3_ and Fe_2_O_3_@TA-Pt NPs as measured in PBS (pH=6.8, 37 ^o^C) B) XPS spectrum of Fe_2_O_3_@TA-Pt NPs. High resolution Pt 4f spectra of Fe_2_O_3_@TA-Pt NPs C) before and D) after treating with H_2_O_2_ under acidic conditions (pH=6.8) for 1 h. High resolution Fe 3d spectra of Fe_2_O_3_@TA-Pt NPs E) before and F) after treating with H_2_O_2_ under acidic conditions (pH=6.8) for 1 h.


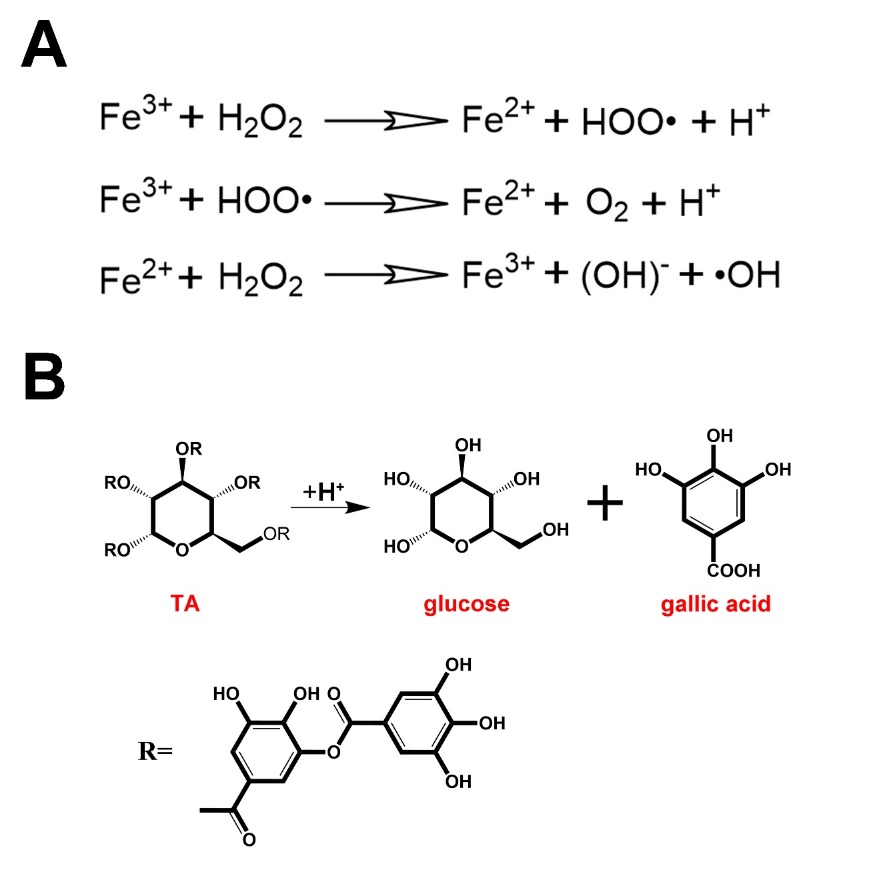


Figure S5. A) Schematic illustration of hydroxyl radical generation. B) Schematic illustration of TA hydrolyzes under acidic conditions.


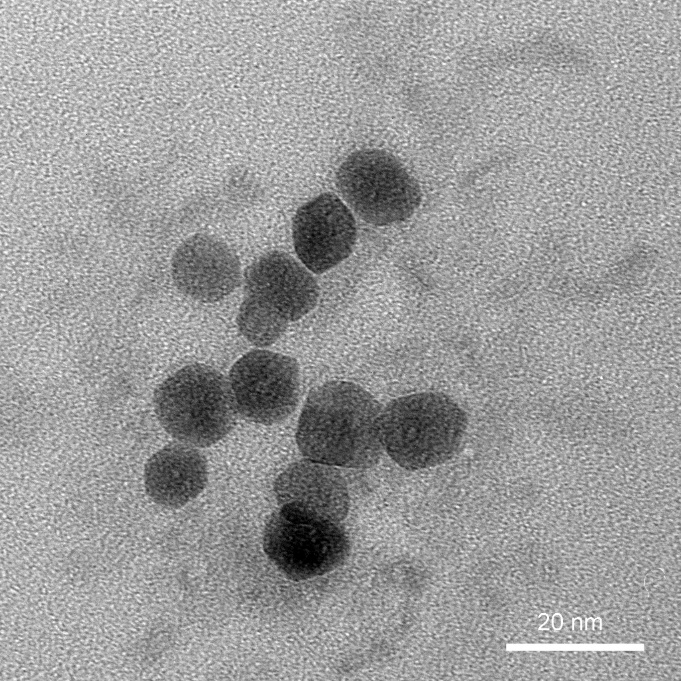


Figure S6. TEM image of Fe_2_O_3_@TA-Pt treated with acidic PBS (pH=6.8) for 24 h.


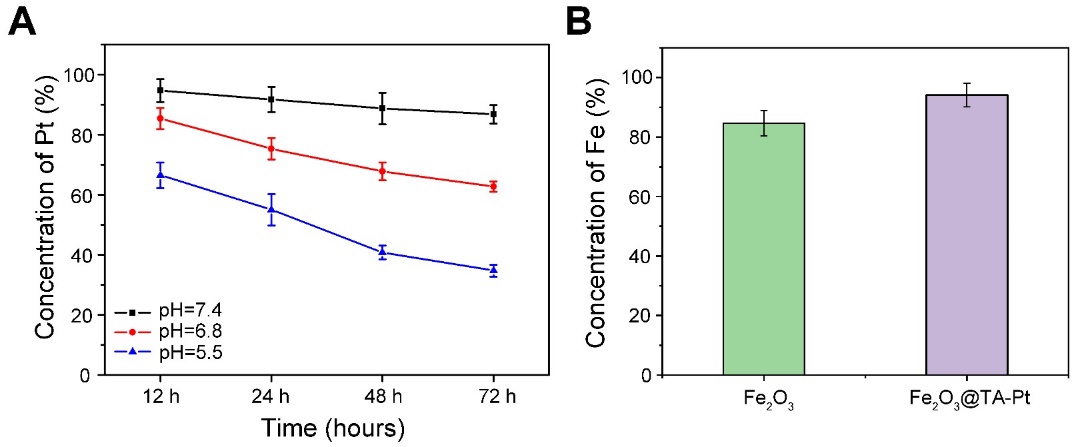


Figure S7. A) The content of Pt in Fe_2_O_3_@TA-Pt NPs cultured with different PBS (pH=7.4, 6.8, 5.5). B) The content of Fe in Fe_2_O_3_ NPs and Fe_2_O_3_@TA-Pt NPs cultured with mouse serum for 72 h.


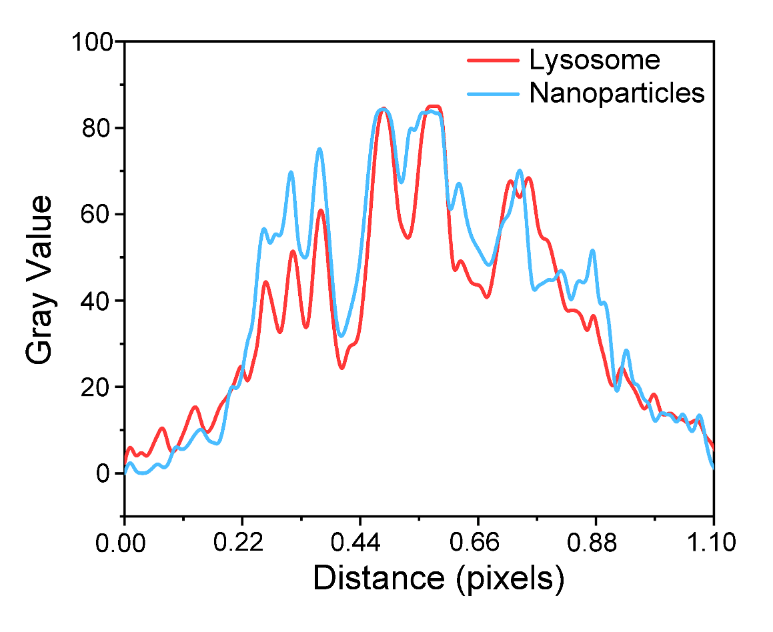


Figure S8. Fluorescence intensity profiles of Fe_2_O_3_@TA-Pt NPs and lysosome were measured using Fiji (Image J) and shown as a function of distance.


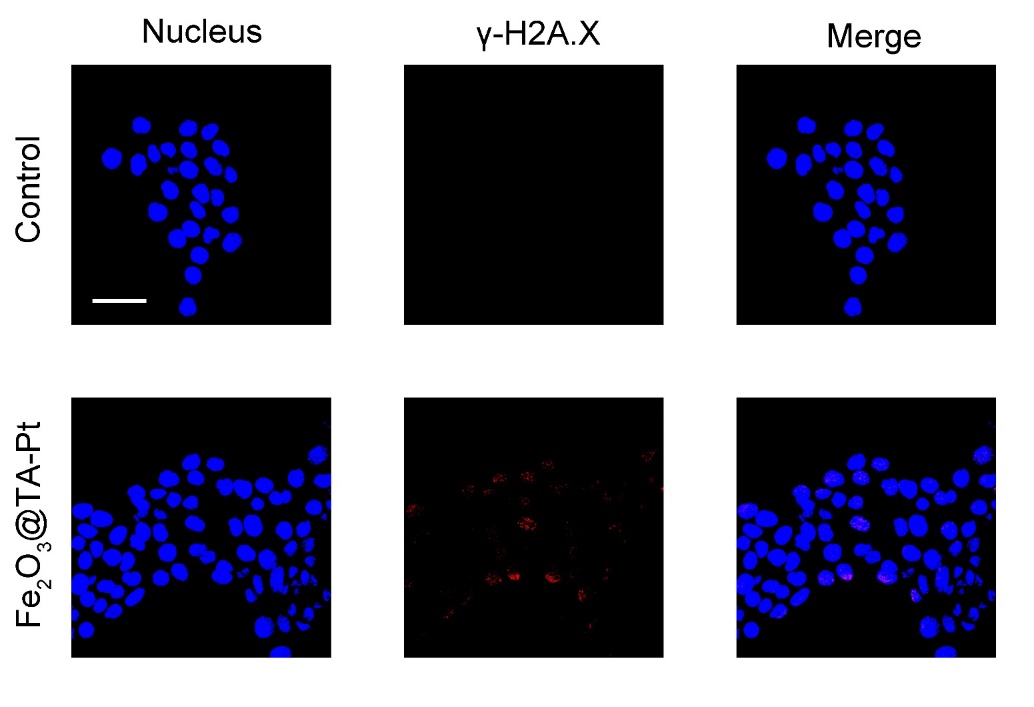


Figure S9. The production of γ-H2A.X in 4T1 cells pre-incubated with NAC to analyze DNA damage caused by TA-Pt film. Scale bars: 10 μm.


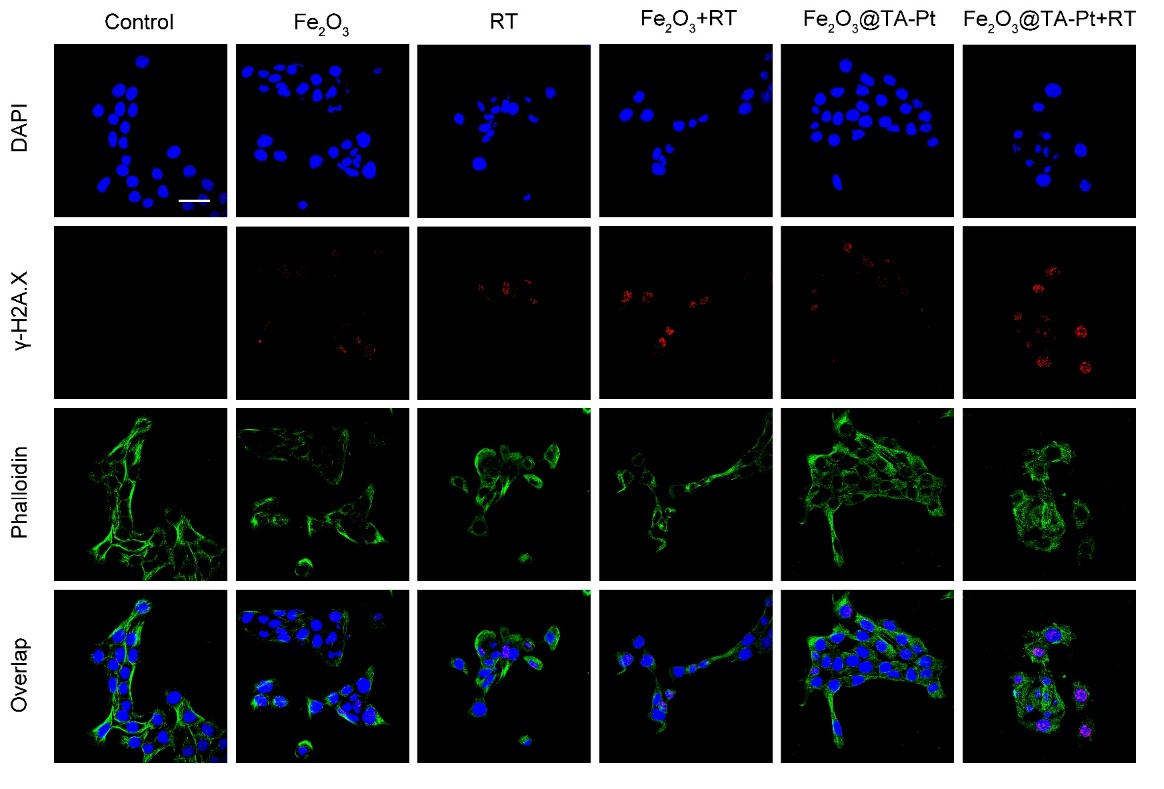


Figure S10. The independent CLSM images of 4T1 cells for testing γ-H2A.X with different treatments (blue: DAPI, green: phalloidin, red: γ-H2A.X). Scale bar: 10 μm.


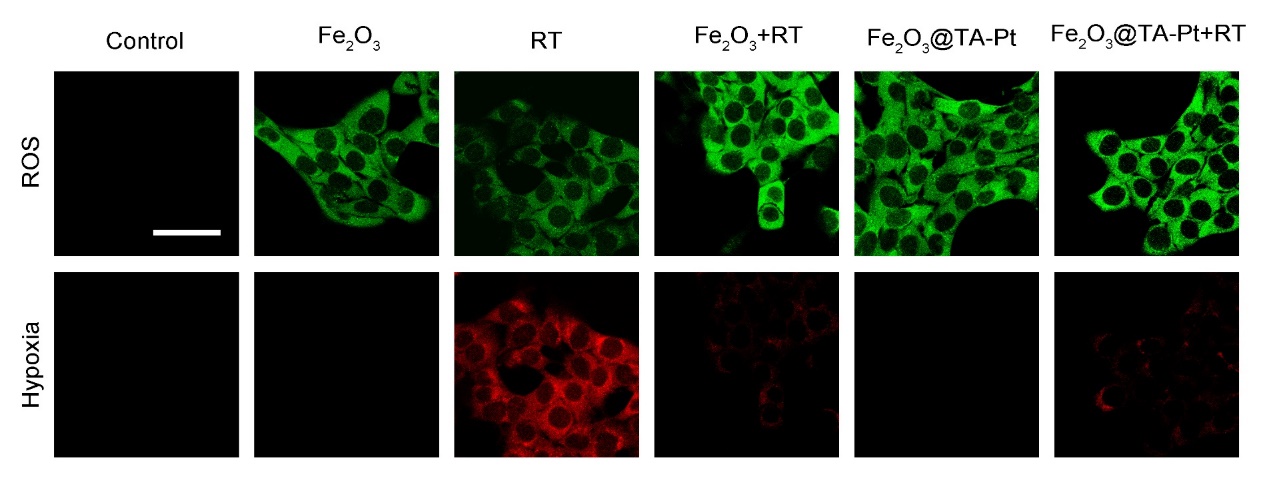


Figure S11. The independent CLSM images of 4T1 cells for ROS/hypoxia detection. Scale bar: 10μm.





Figure S12. *In vitro* viability of 4T1 cells pre-incubated with NAC to analyze the


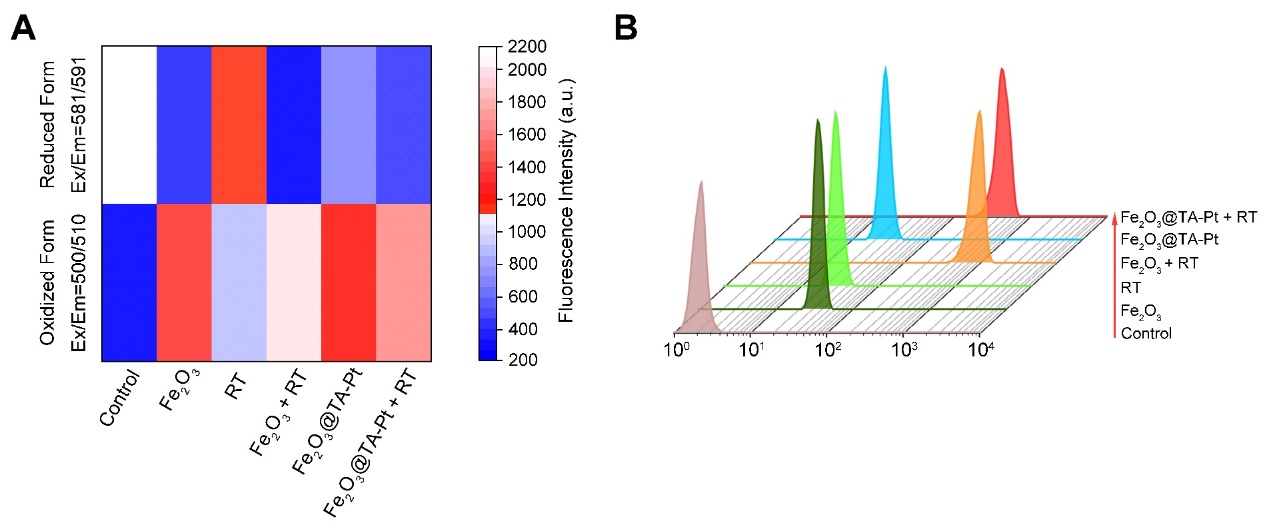


Figure S13. A) Flow cytometry of LPO levels after cells were treated with different types of treatments as displayed. B) The fluorescence intensities of ROS generated in 4T1 cells during different types of treatments and measured by flow cytometry.


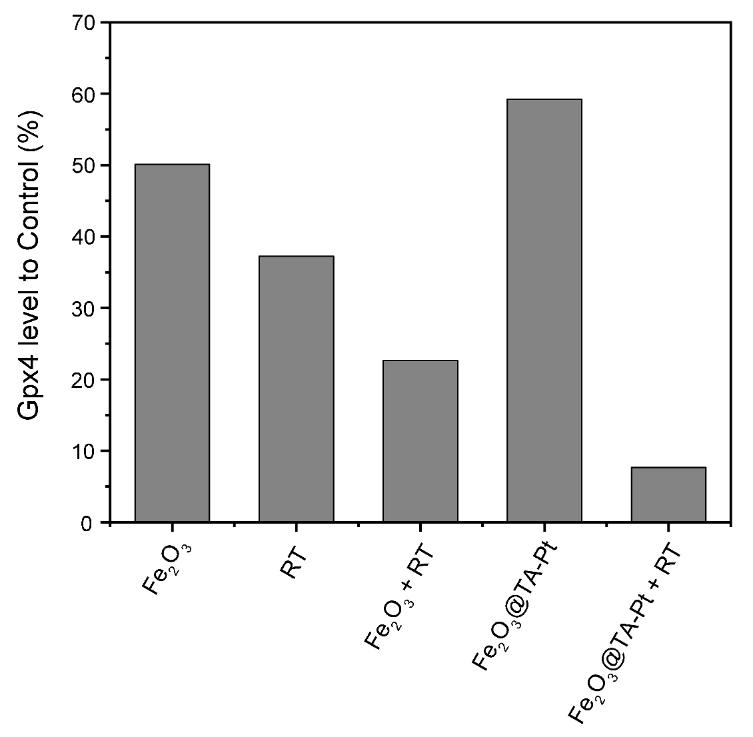


Figure S14. The gray ratio of western blot analysis (GPX4) to the control group.


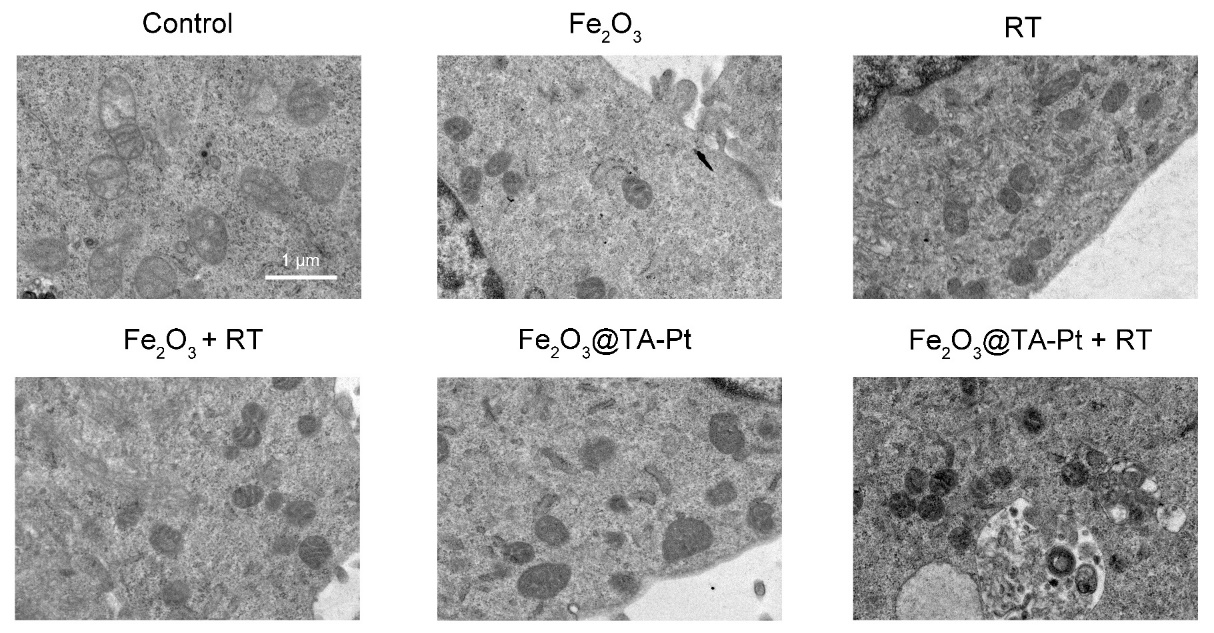


Figure S15. Cells morphology with Fe_2_O_3_@TA-Pt-mediated ferroptosis after incubated with NPs or irradiation for 24h.


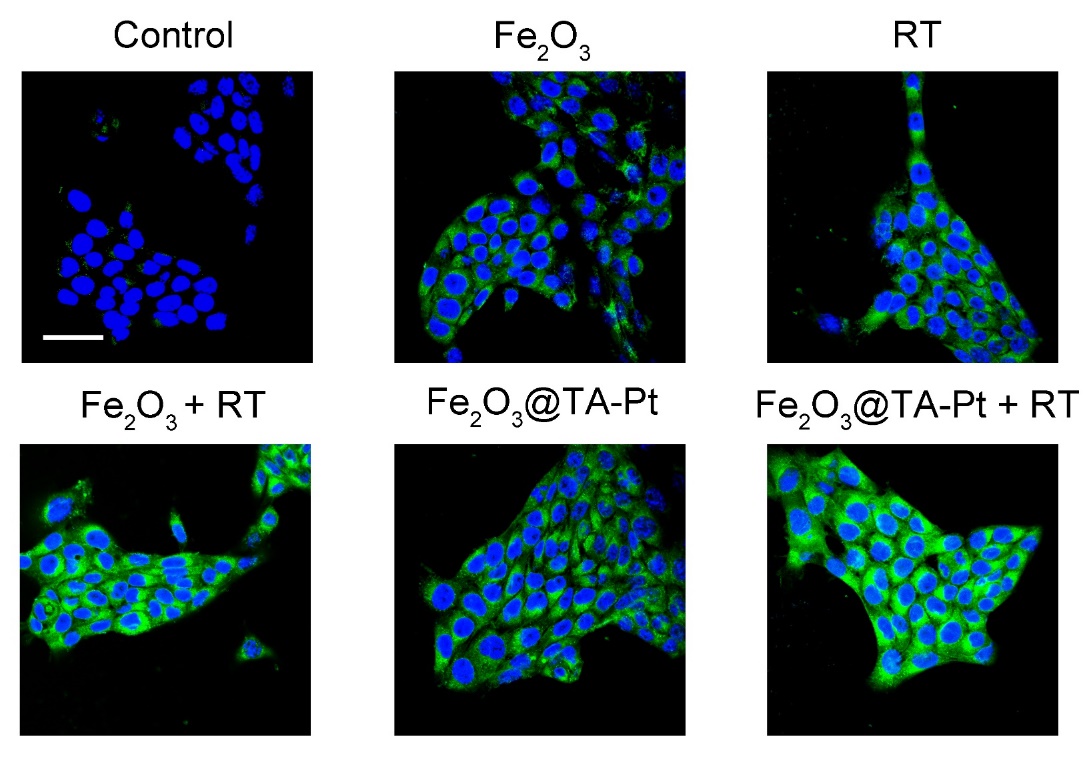


Figure S16. Cleaved Caspase-3 analysis of 4T1 cells subjected to different treatments. Scale bars: 10 μm.


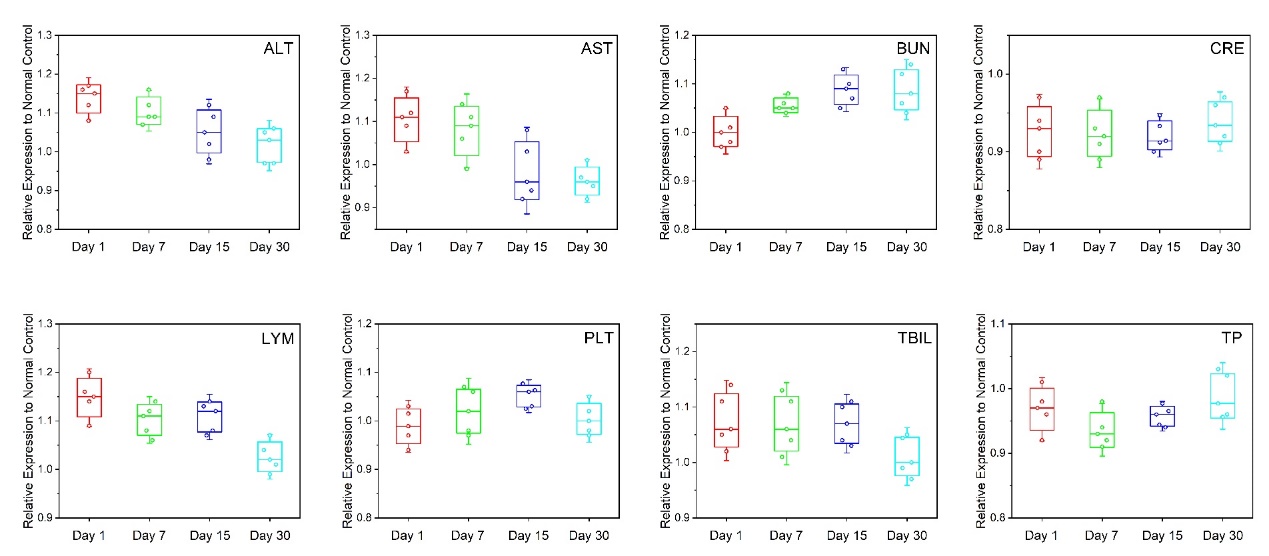


Figure S17. The hematology assay of 4T1-tumor-bearing mice with Fe_2_O_3_@TA-Pt under radiotherapy at days 1, 7, 15, and 30.


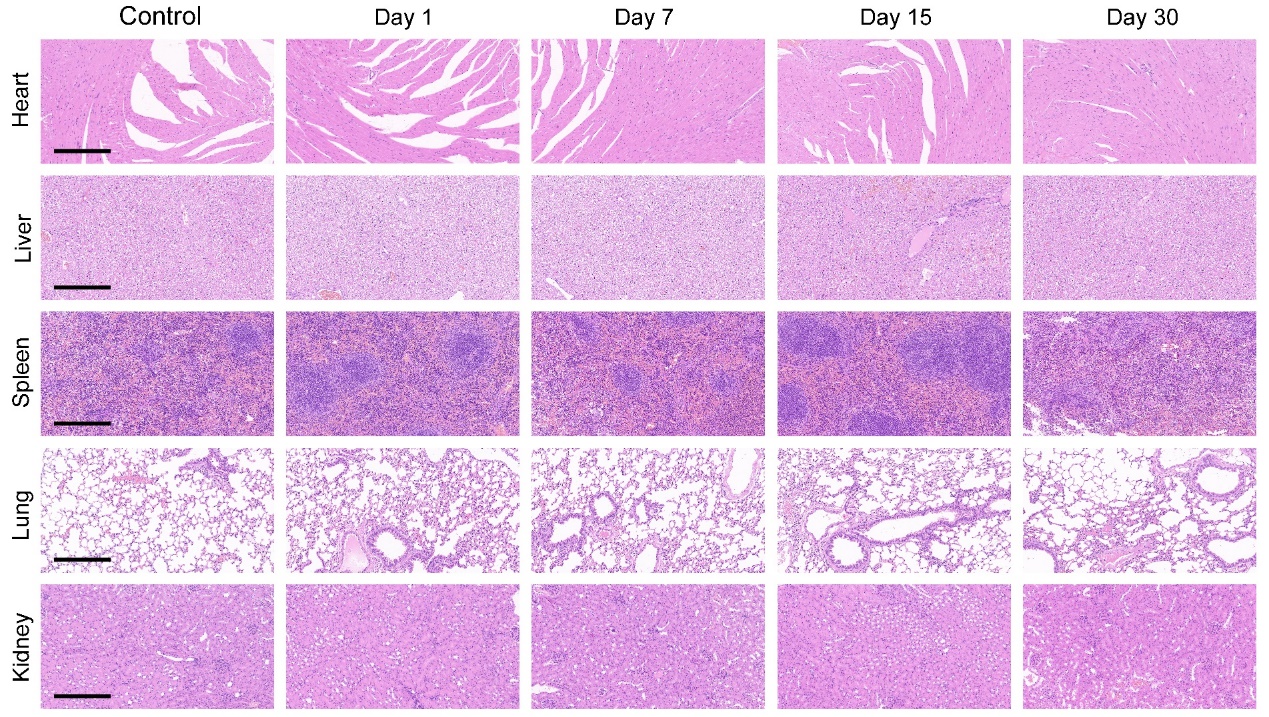


Figure S18. The long-term toxicity of Fe_2_O_3_@TA-Pt in heart, liver, spleen, lung, and kidney at days 1, 7, 15, and 30 post intravenous injection of Fe_2_O_3_@TA-Pt NPs using H&E staining. Scale bars: 100 μm.
